# Supplementary material for: An epigenome-wide analysis of DNA methylation, racialized and economic inequities, and air pollution
Source: bioRxiv. 2023 Dec 8:2023.12.07.570610. Preprint. [Version 1] doi: 10.1101/2023.12.07.570610 (PMC10723401; doi:10.1101/2023.12.07.570610)

# Supplementary figures

## Miami plots

#
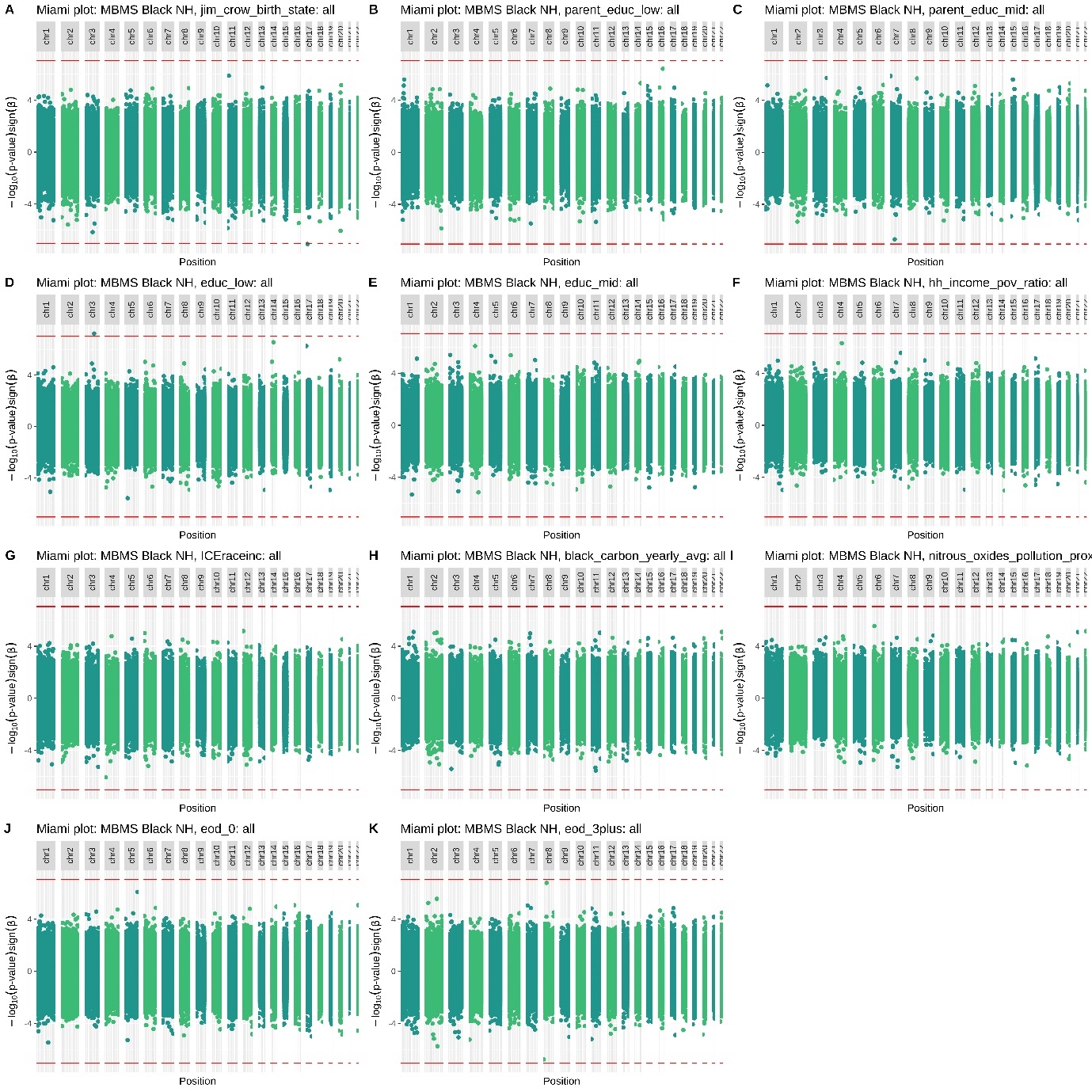


Figure 1: MBMS Black NH Miami plots

#
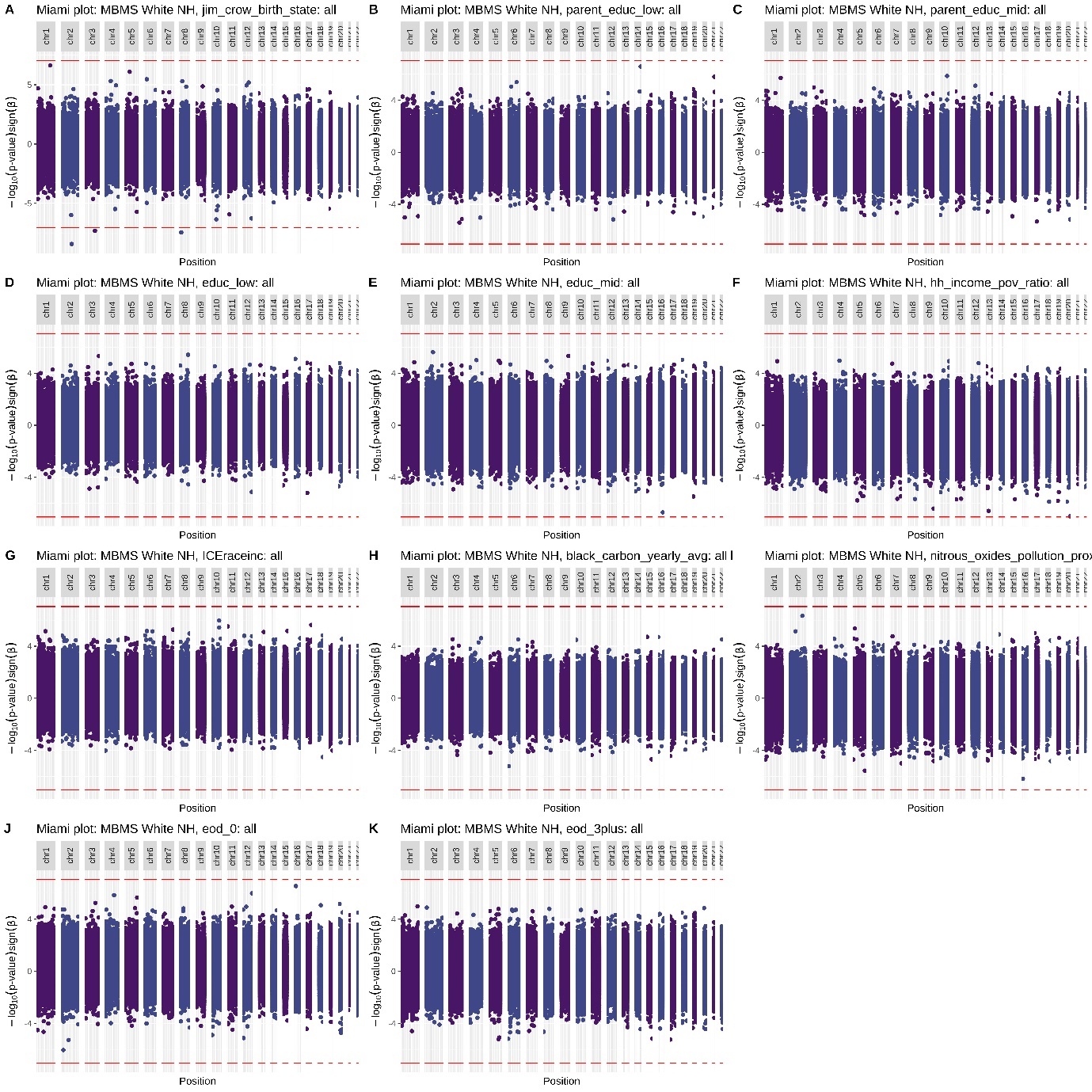


Figure 2: MBMS white NH Miami plots


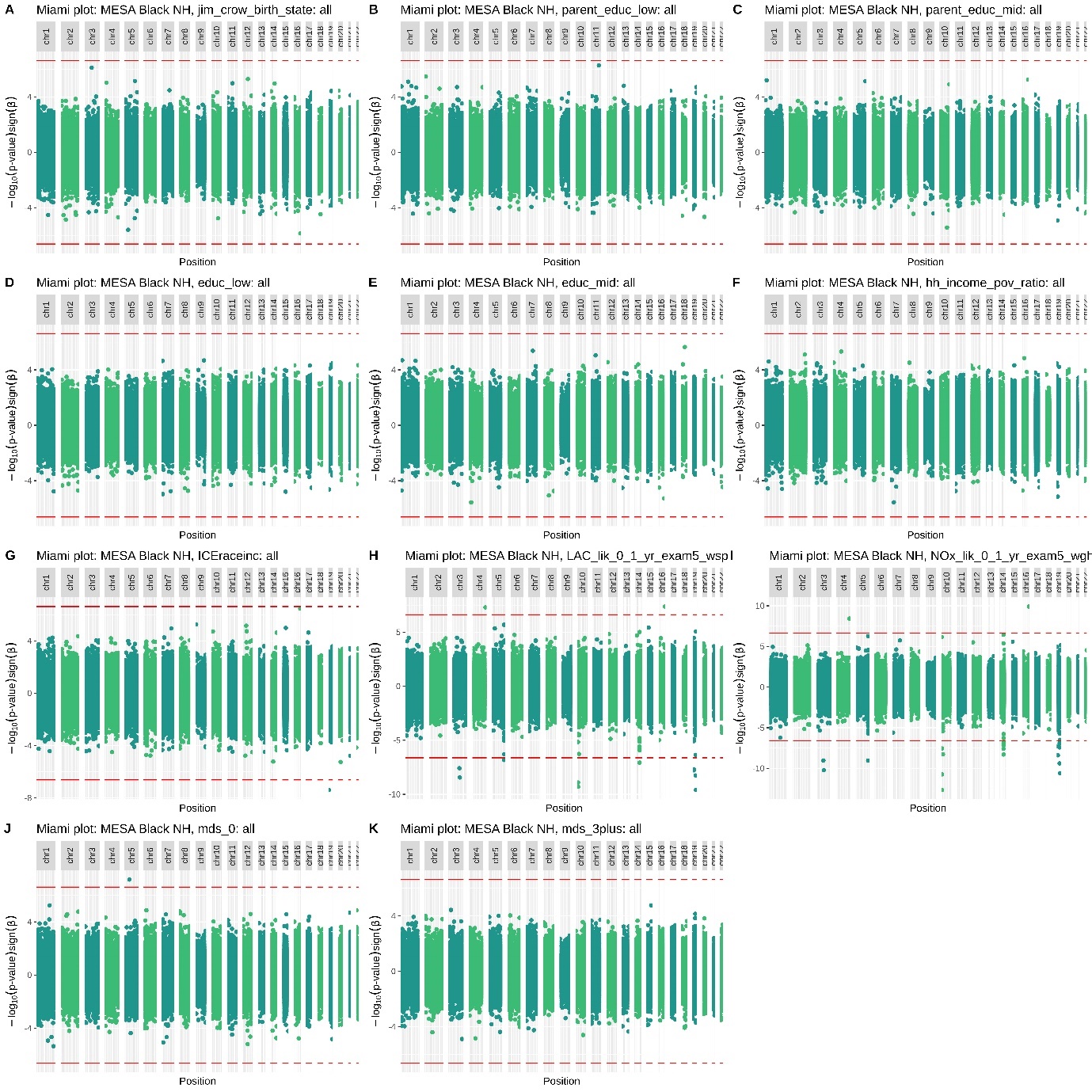


Figure 3: MESA Black NH Miami plots


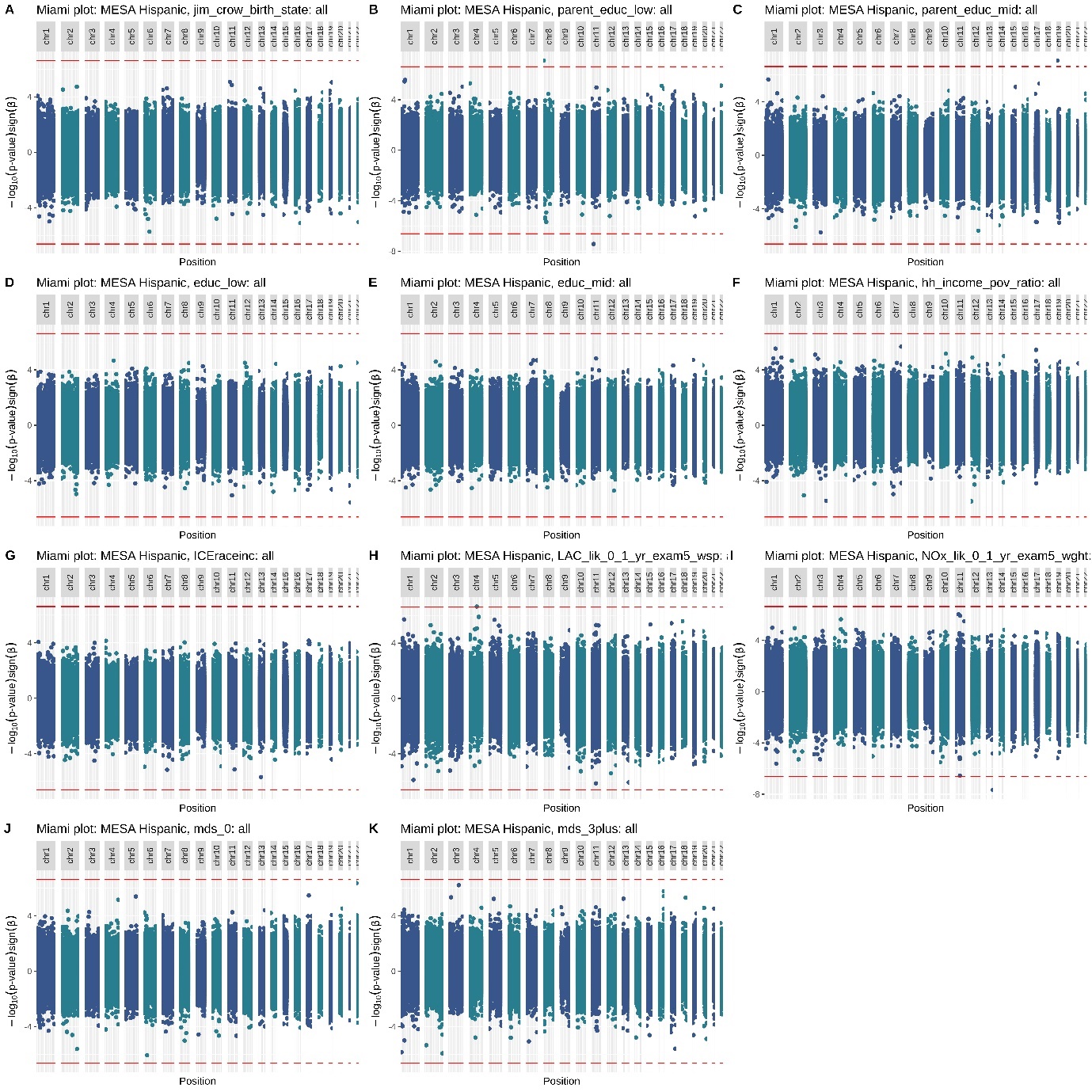


Figure 4: MESA Hispanic Miami plots


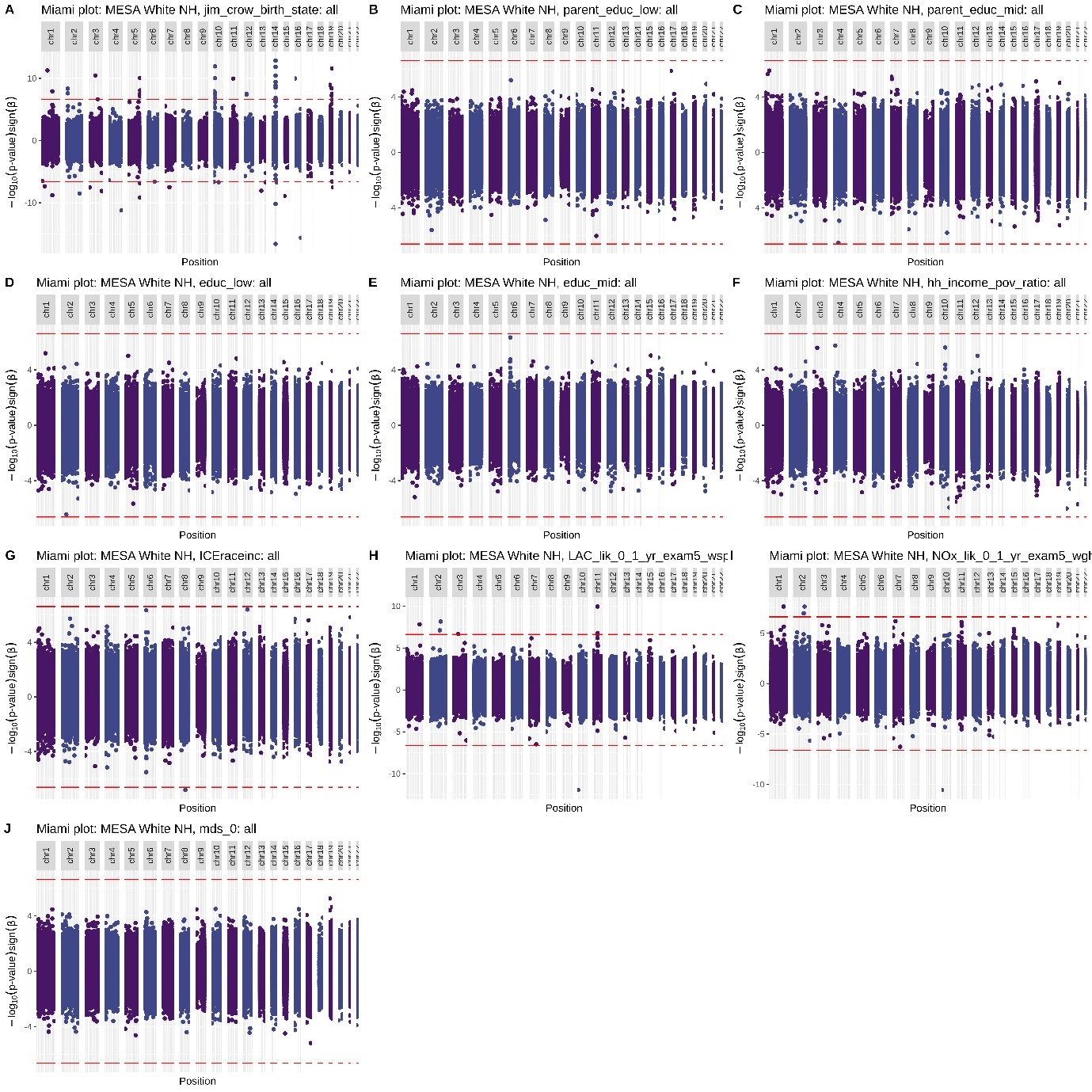


Figure 5: MESA white NH Miami plots

## MESA New York and Baltimore subset Miami plots


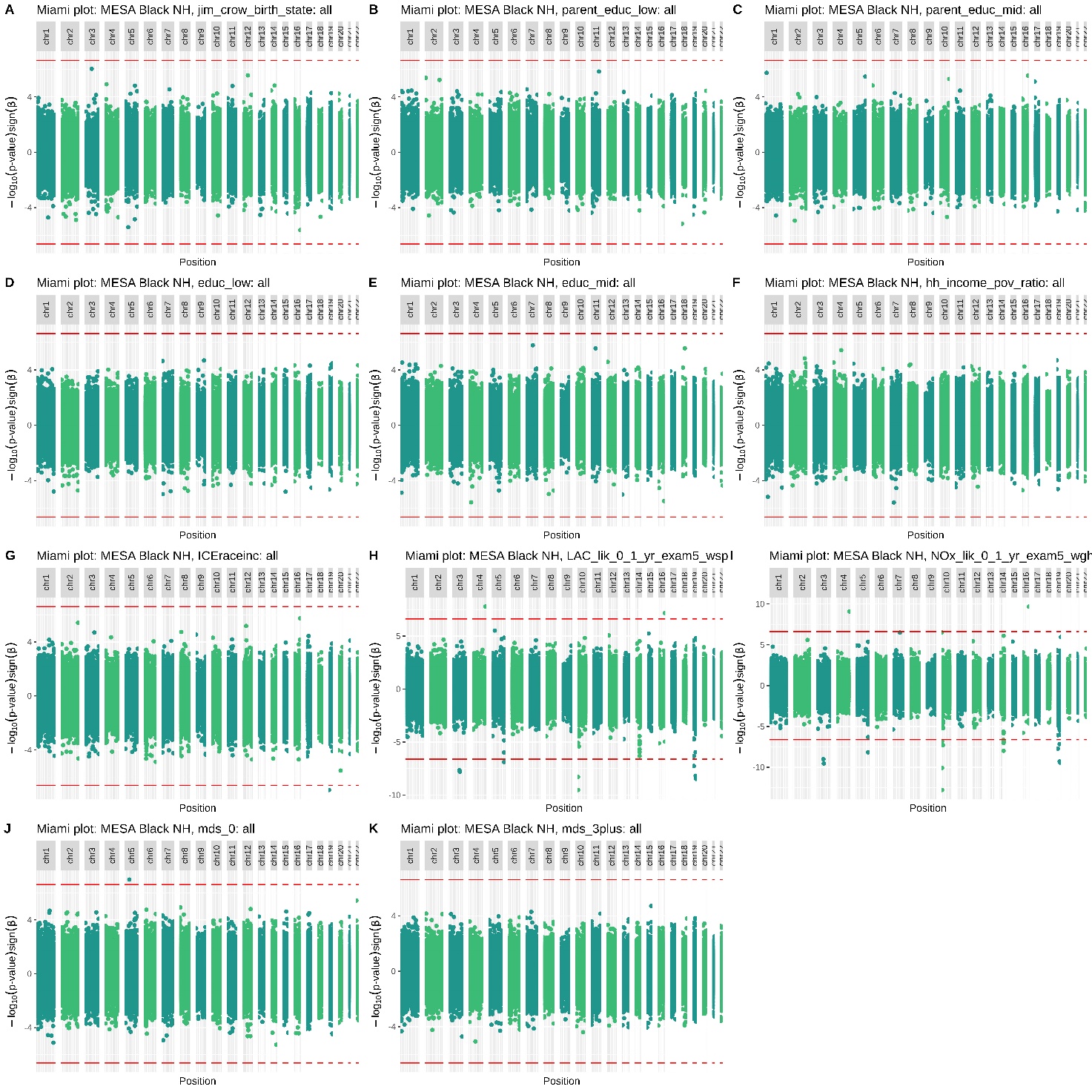


Figure 6: MESA Black NH Miami plots (JHU and COL subgroup)


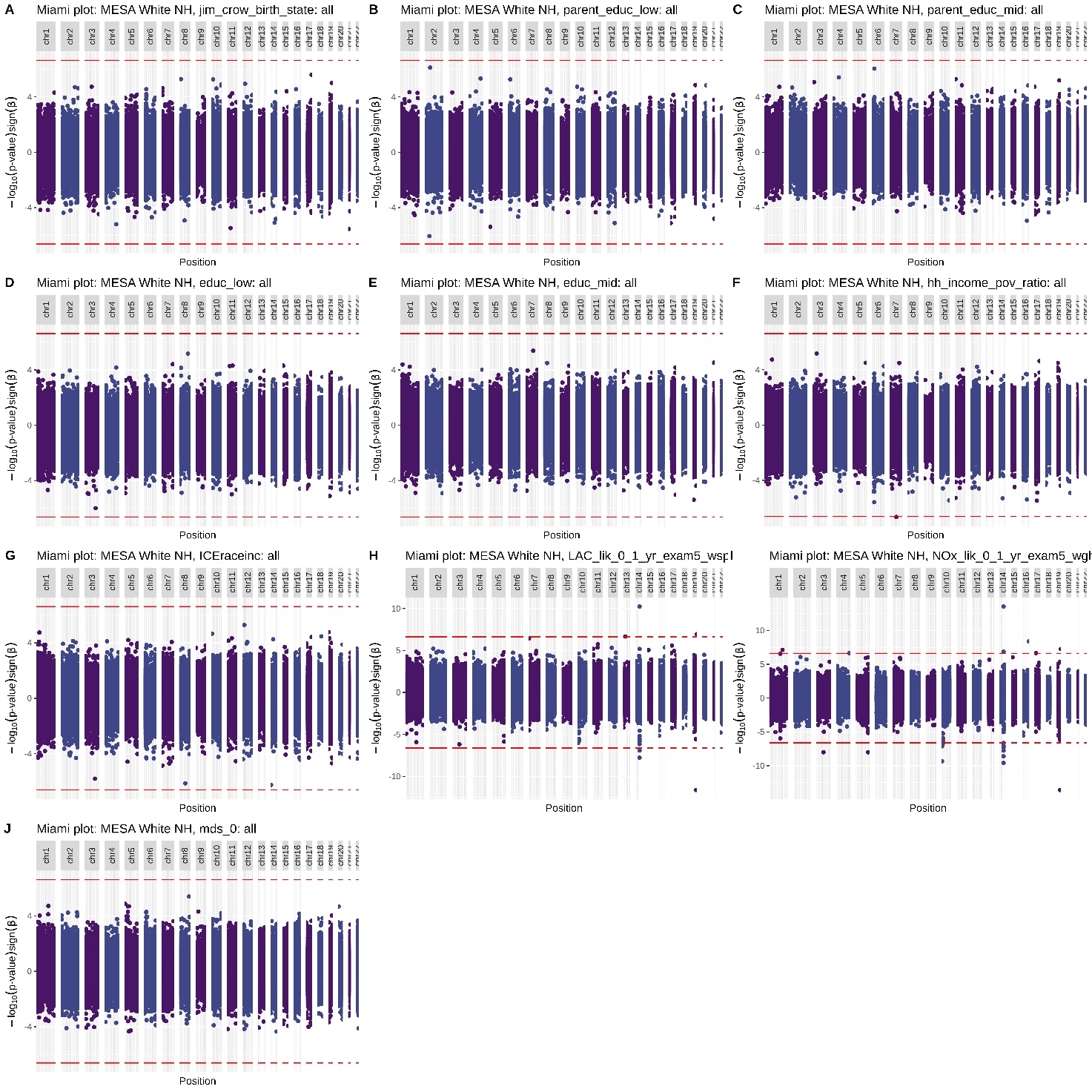


Figure 7: MESA white NH Miami plots (JHU and COL subgroup)

Figure 8: MESA white NH Miami plots (JHU and COL subgroup)

## EWAS catalog enrichment plots

### MBMS full cohort


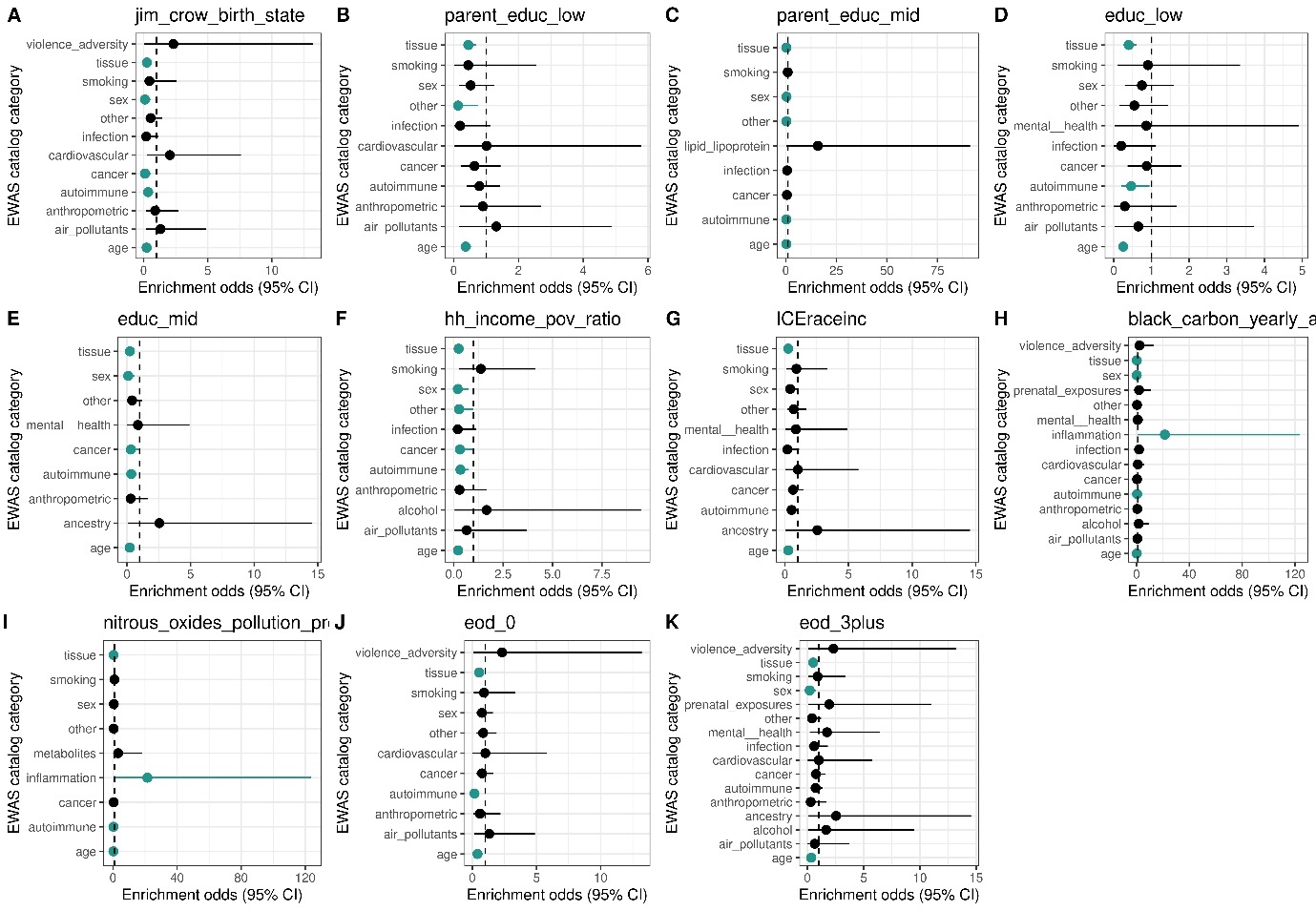


Figure 9: EWAS catalog enrichment plot: MBMS Black NH

----------------------------------------------------------------------


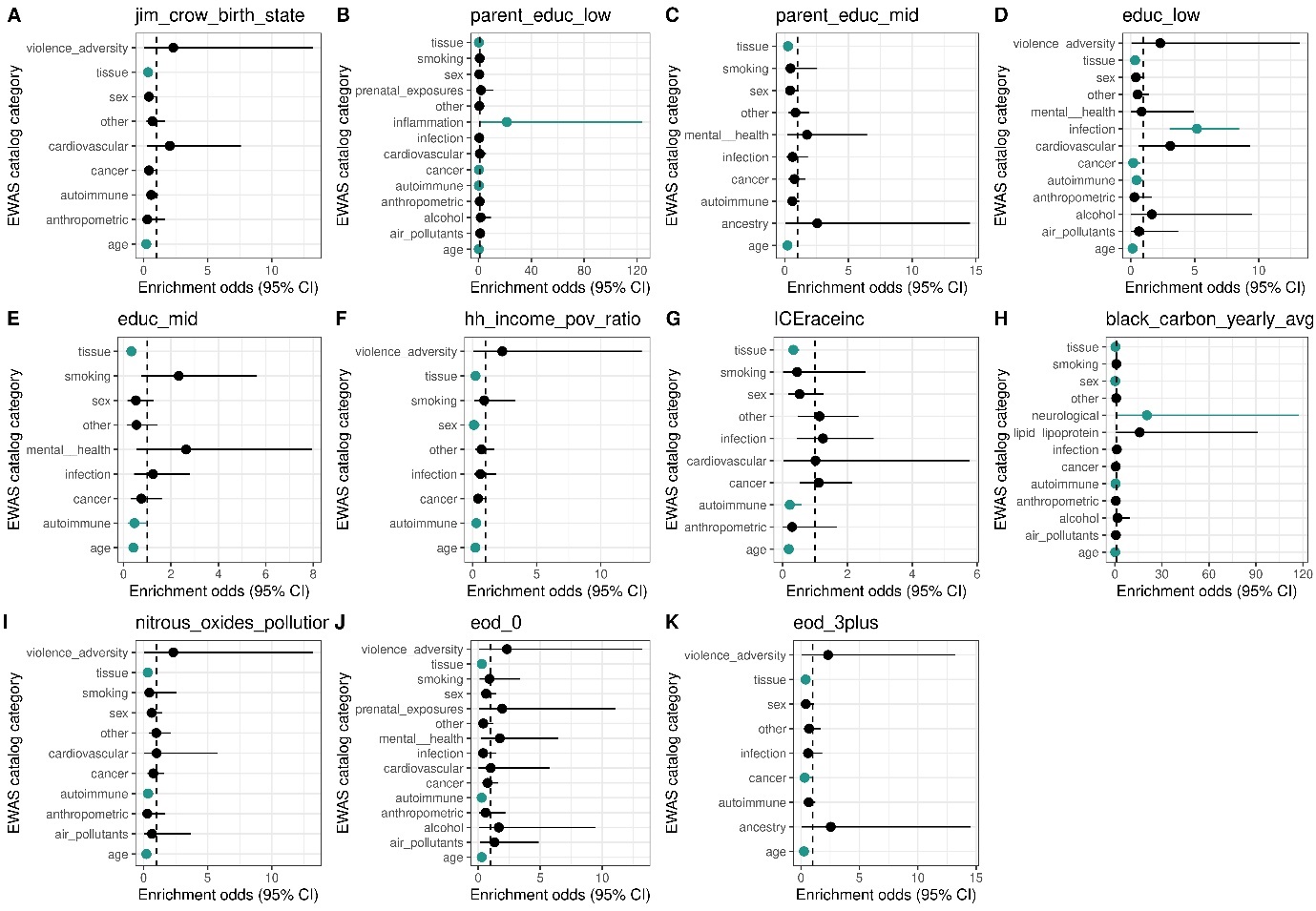


Figure 10: EWAS catalog enrichment plot: MBMS white NH

----------------------------------------------------------------------

### MESA full cohort


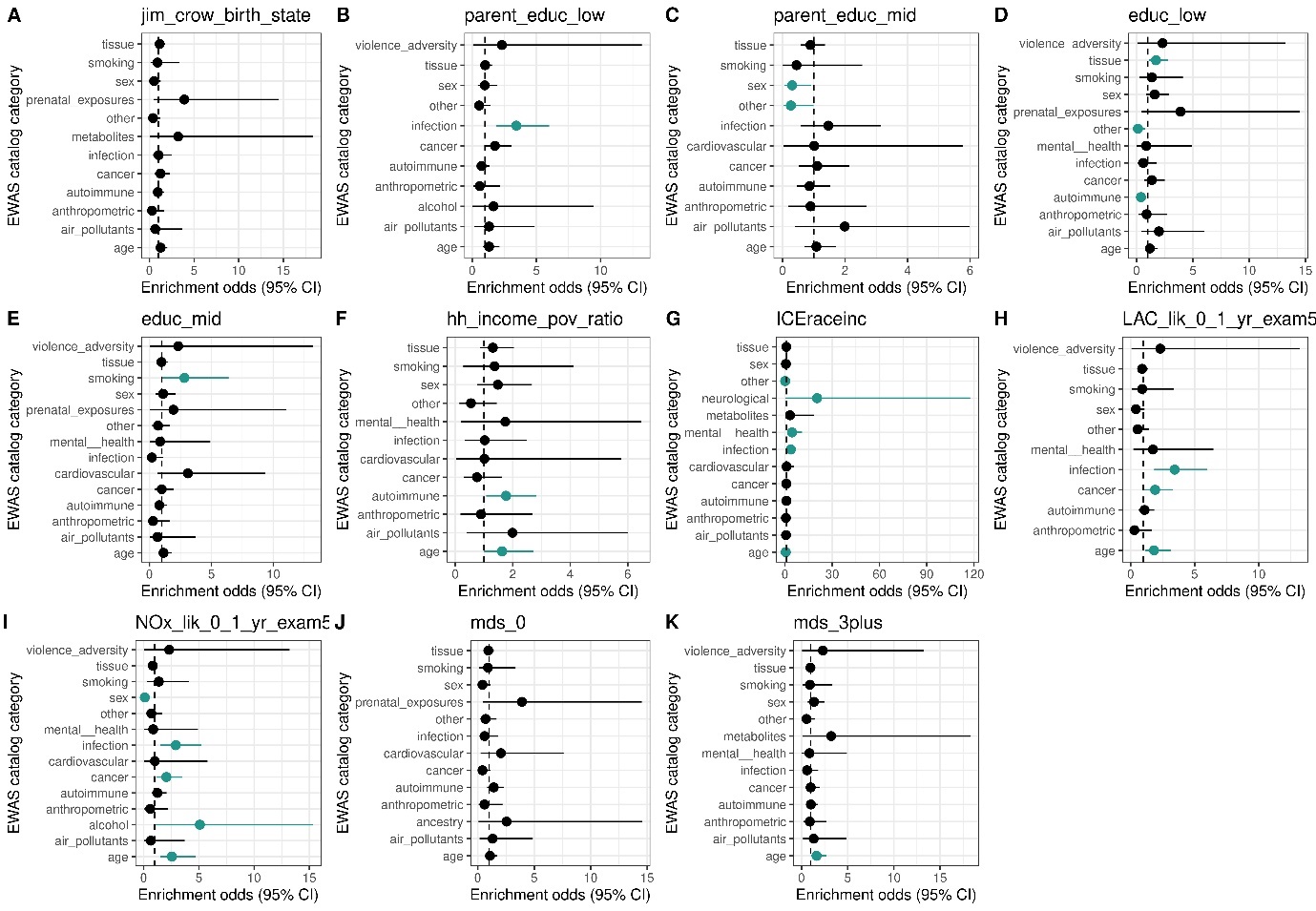


Figure 11: EWAS catalog enrichment plot: MESA Black NH

----------------------------------------------------------------------


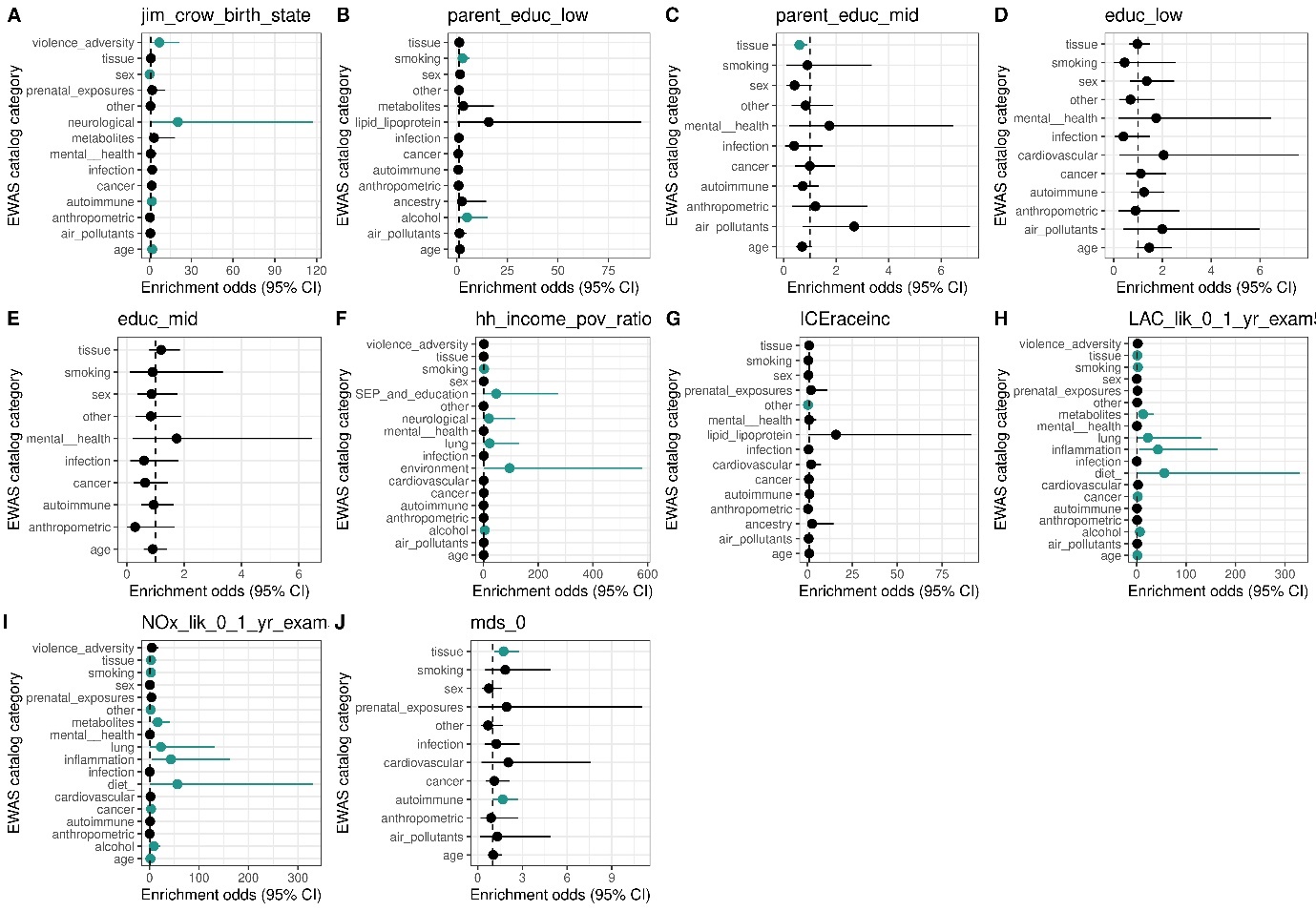


Figure 12: EWAS catalog enrichment plot: MESA white NH


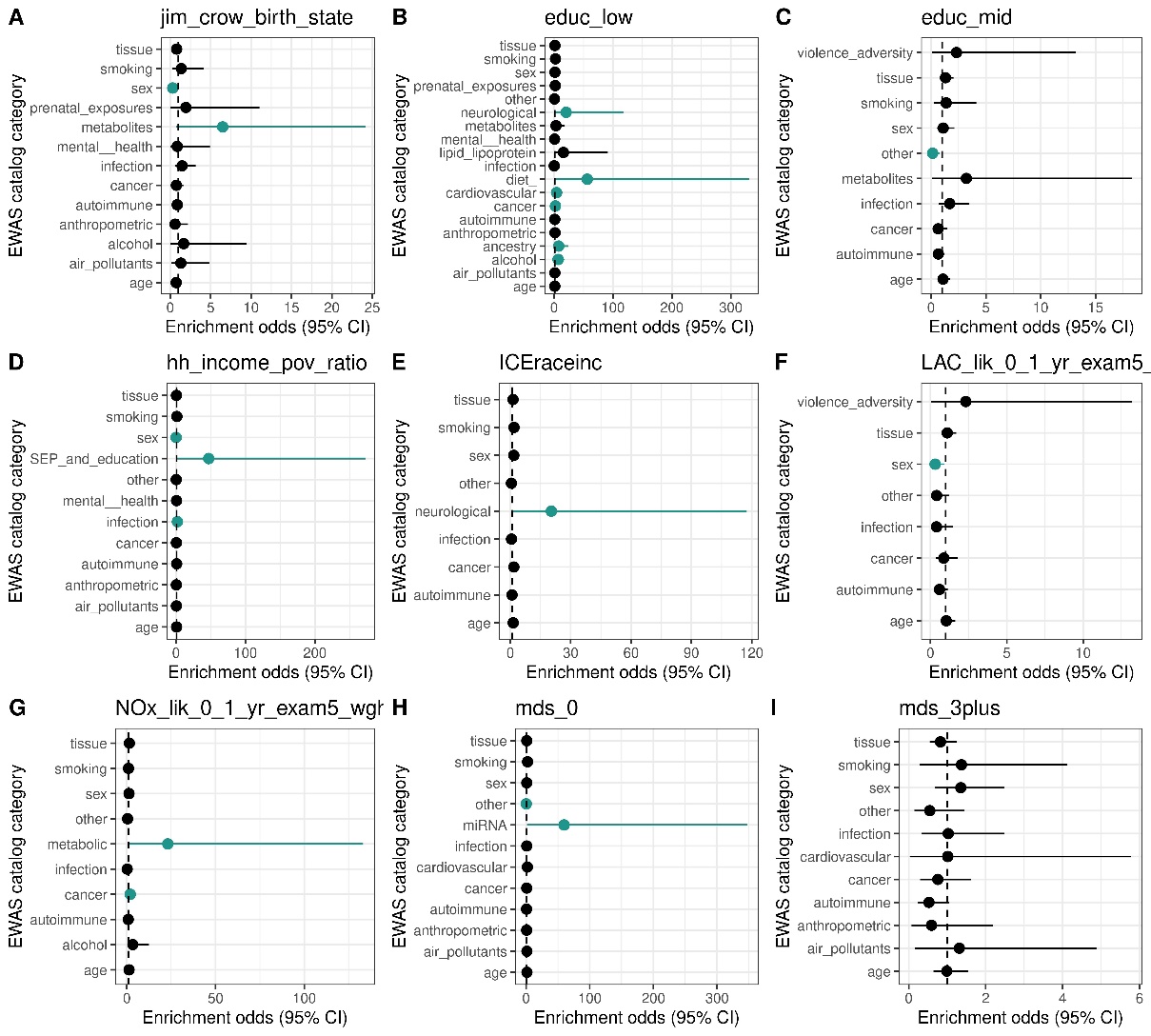


Figure 13: EWAS catalog enrichment plot: MESA Hispanic

----------------------------------------------------------------------

### MESA JHU/COL subgroup


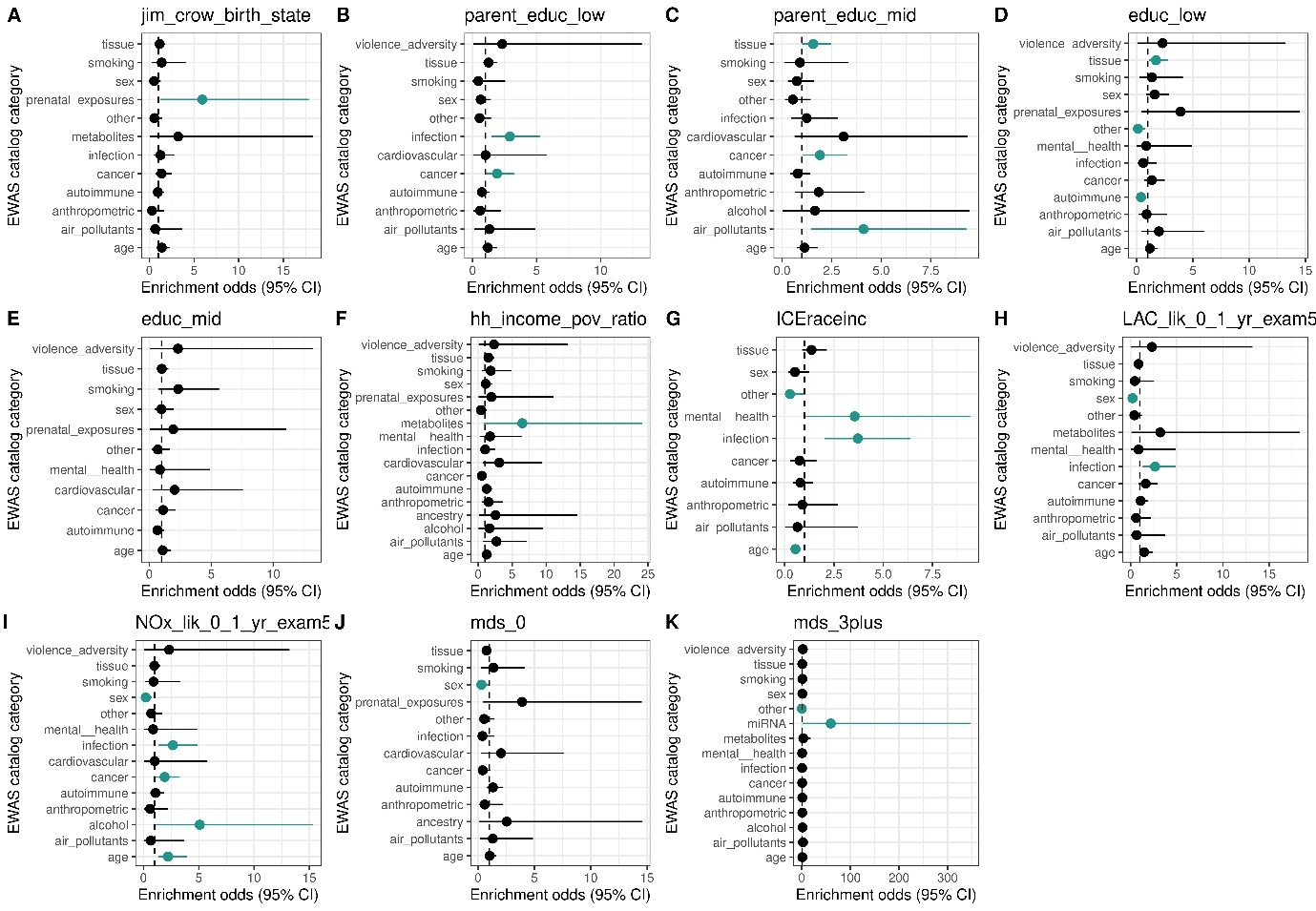


Figure 14: EWAS catalog enrichment plot: MESA Black NH JHU + COL subgroup


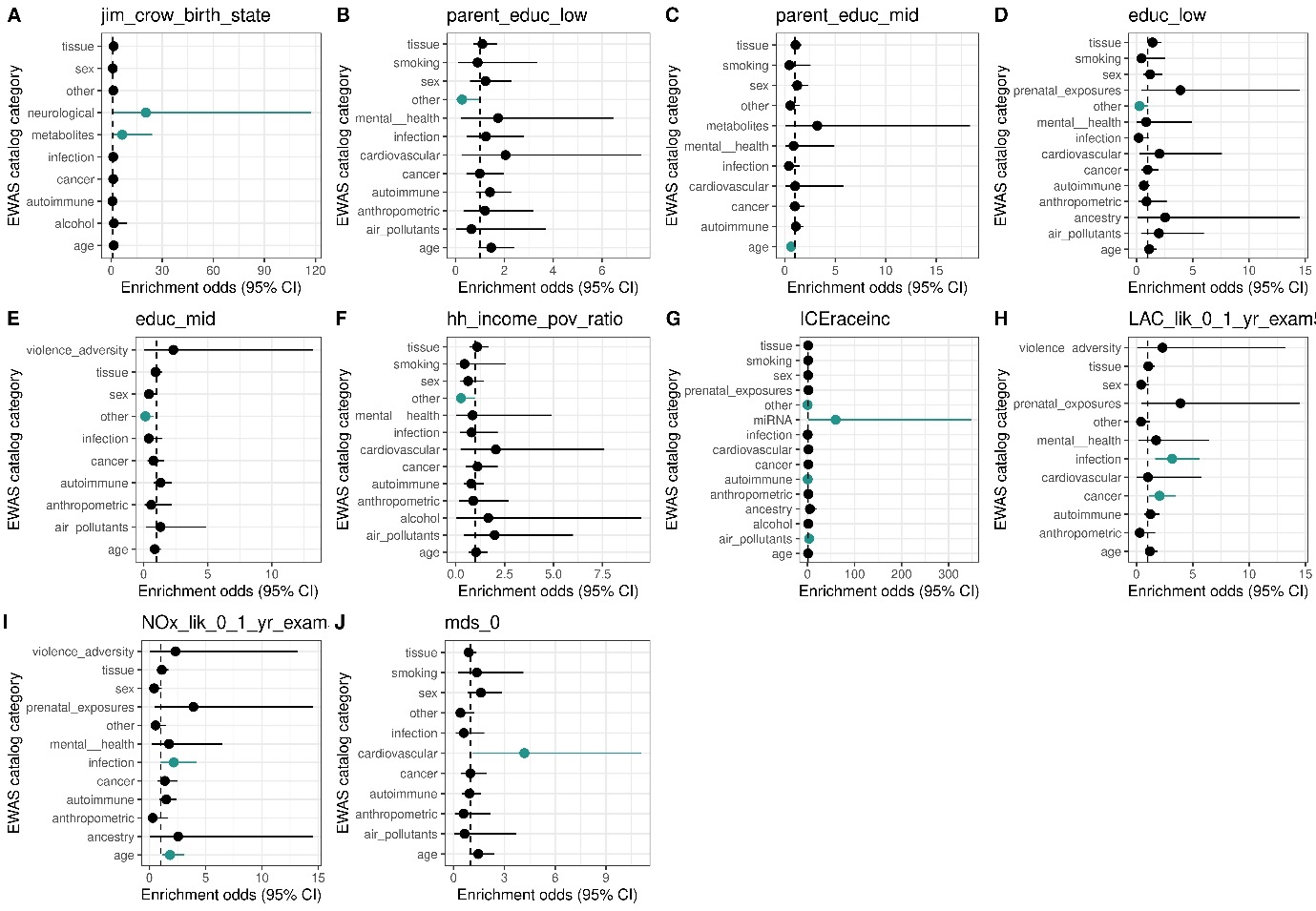


Figure 15: EWAS catalog enrichment plot: MESA white NH JHU + COL subgroup

----------------------------------------------------------------------


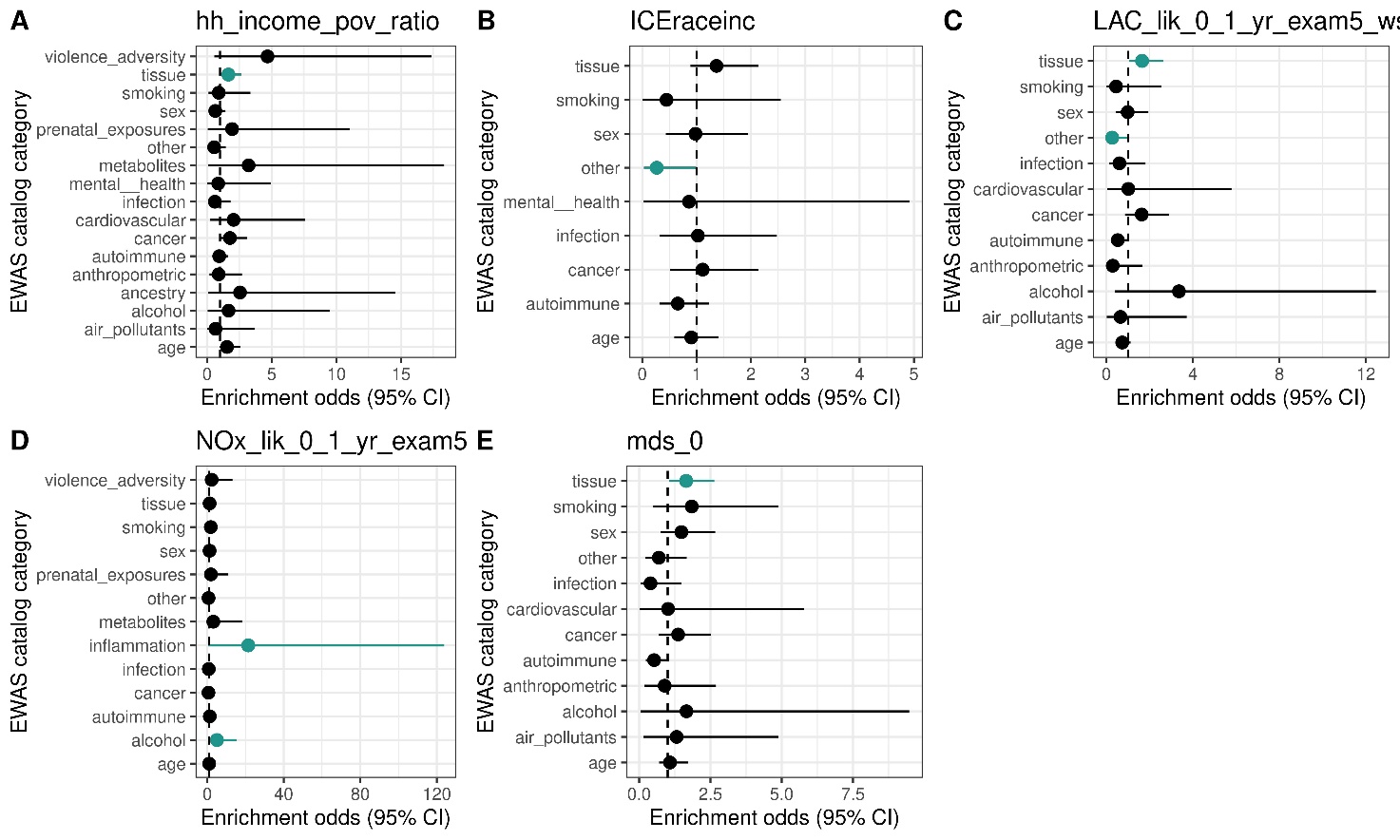


Figure 16: EWAS catalog enrichment plot: MESA Hispanic JHU + COL subgroup


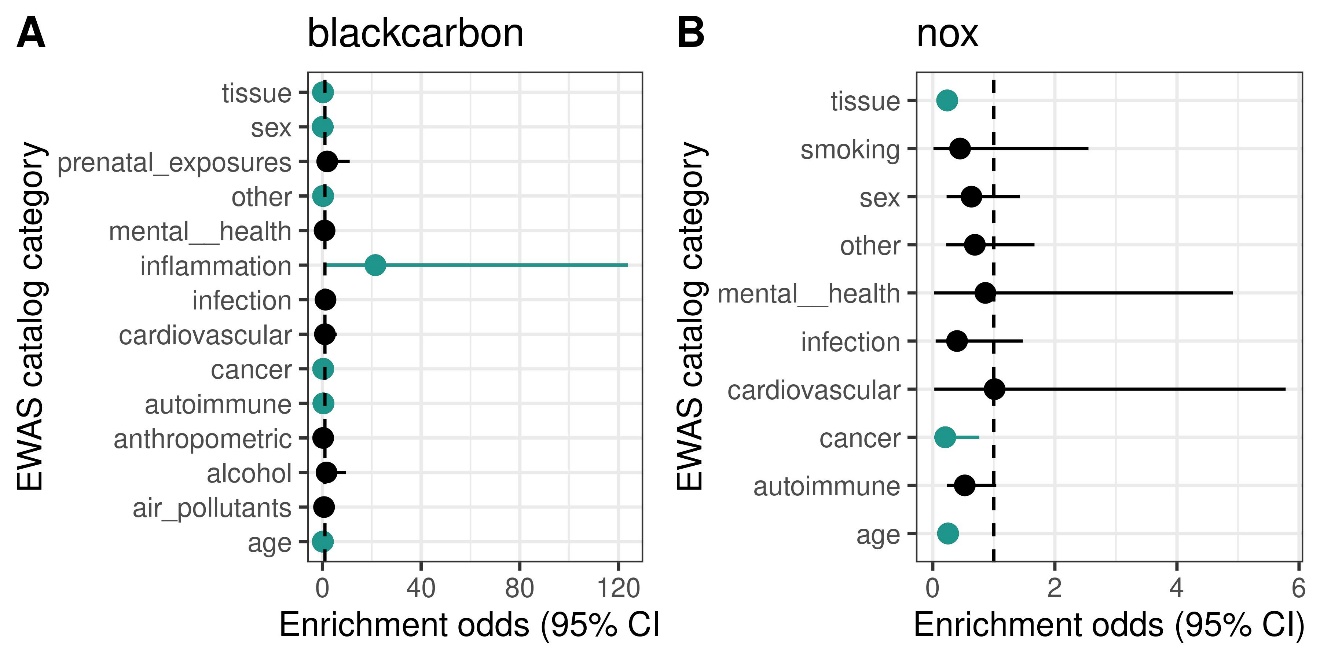


Figure 17: EWAS catalog enrichment plot: MBMS meta-analysis


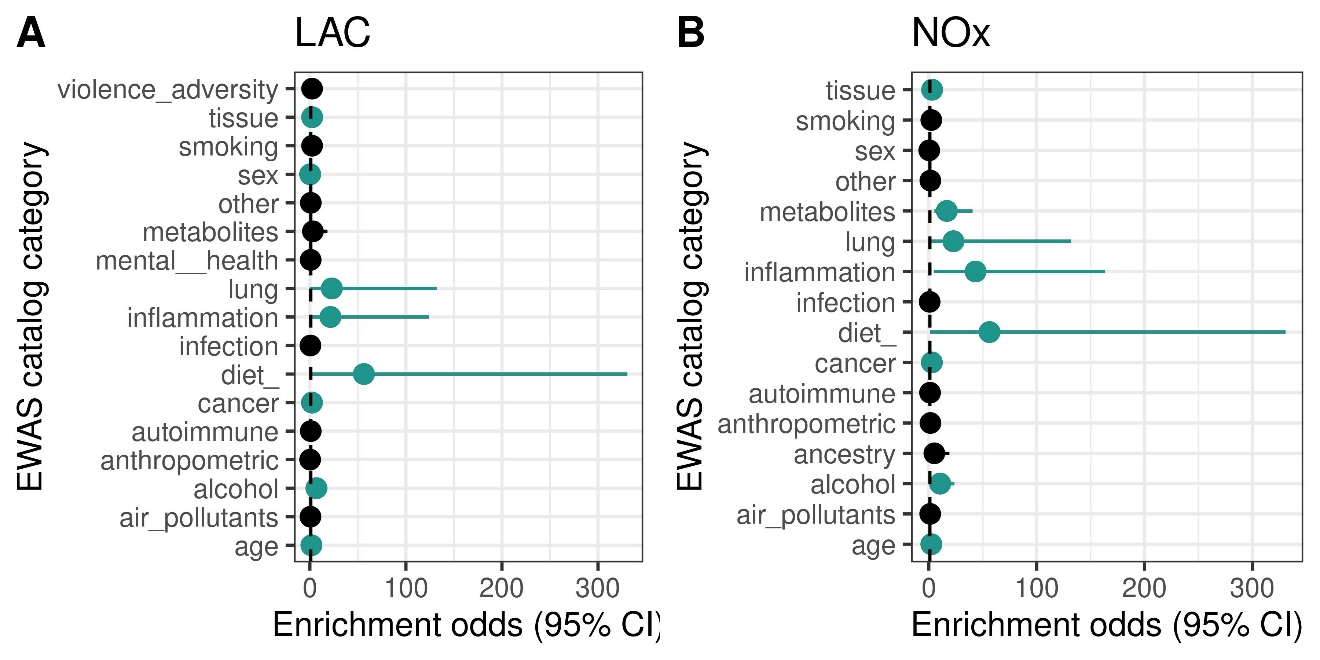


Figure 18: EWAS catalog enrichment plot: MESA meta-analysis


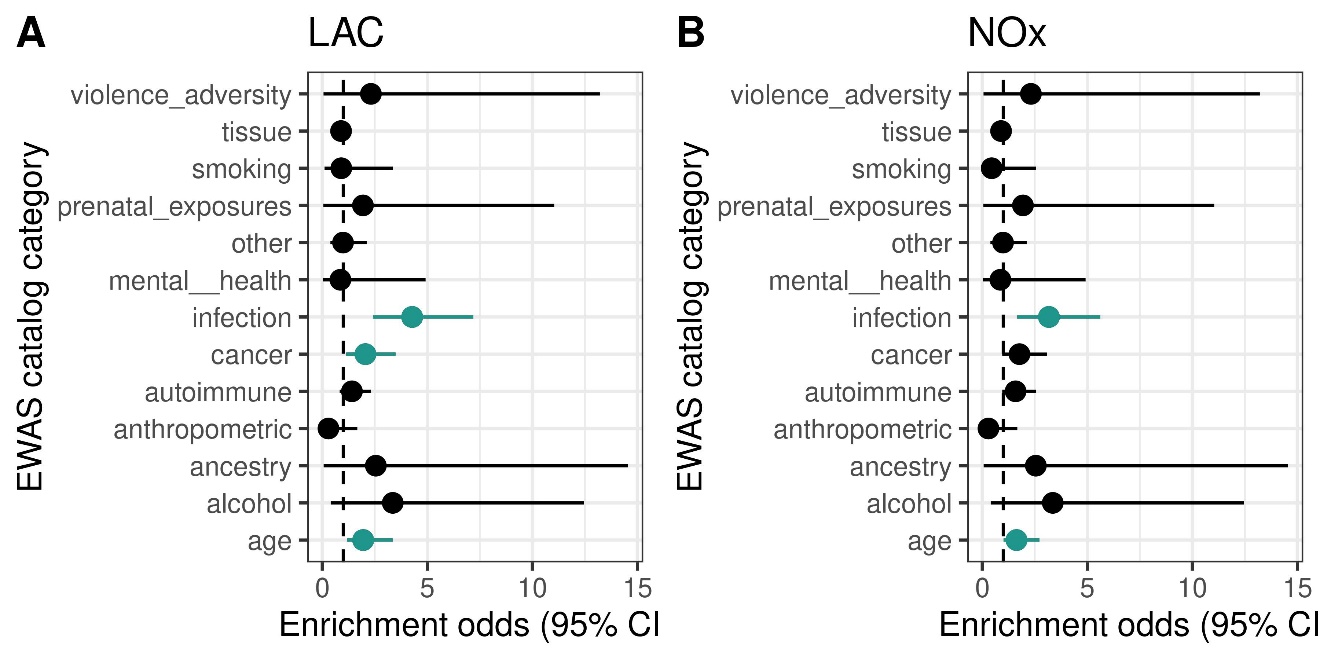


Figure 19: EWAS catalog enrichment plot: MESA meta-analysis (JHU and COL subgroup)

## Enrichment for genomic features

### MBMS full cohort


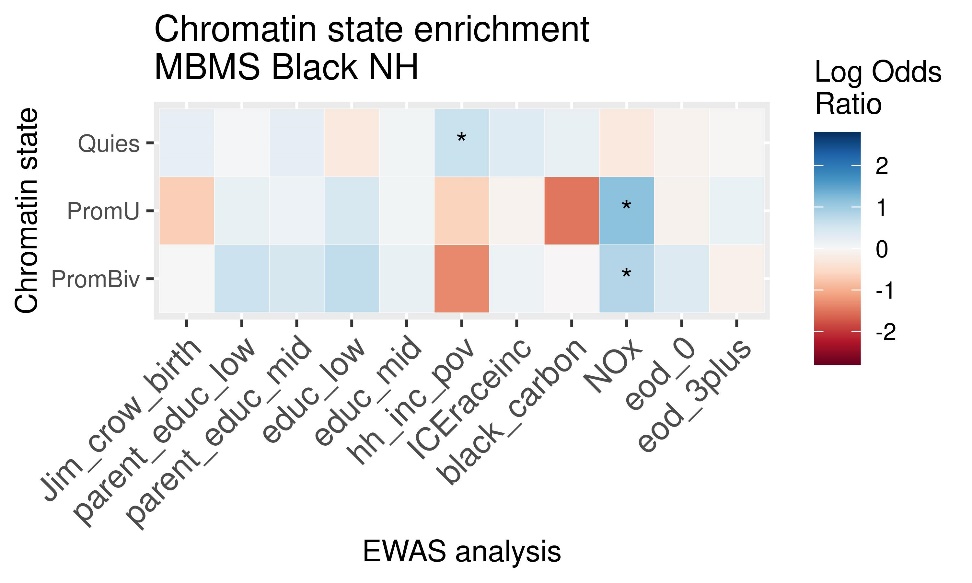


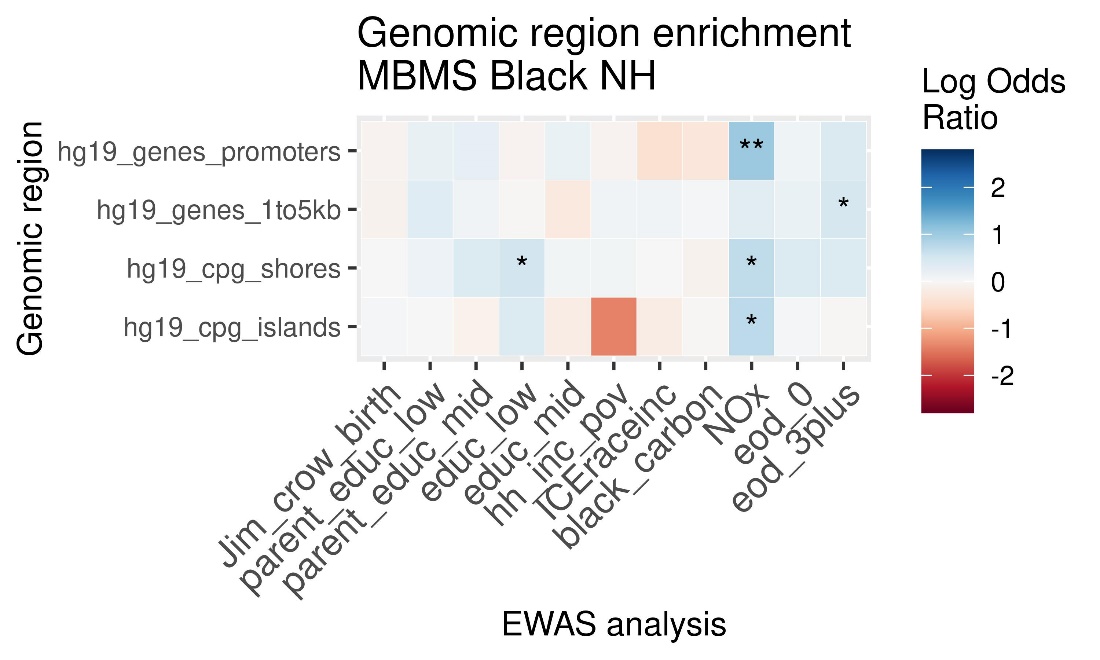


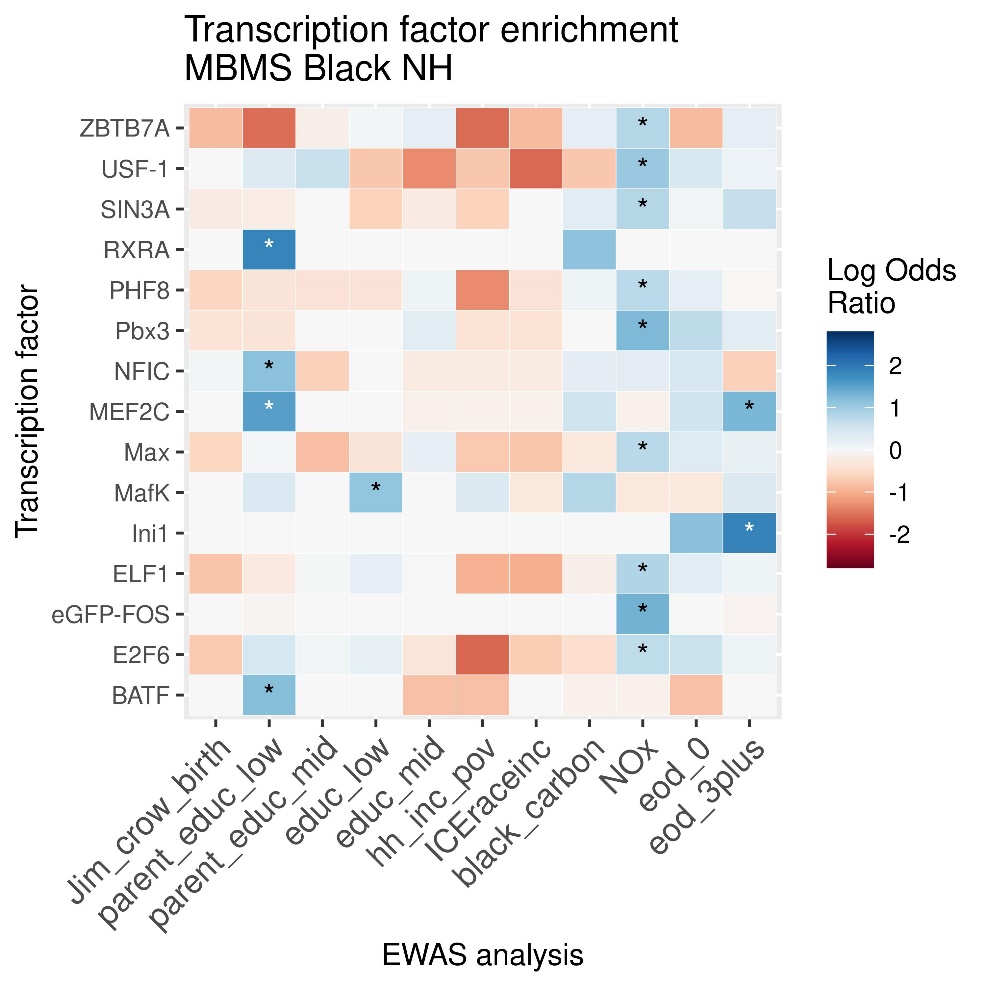


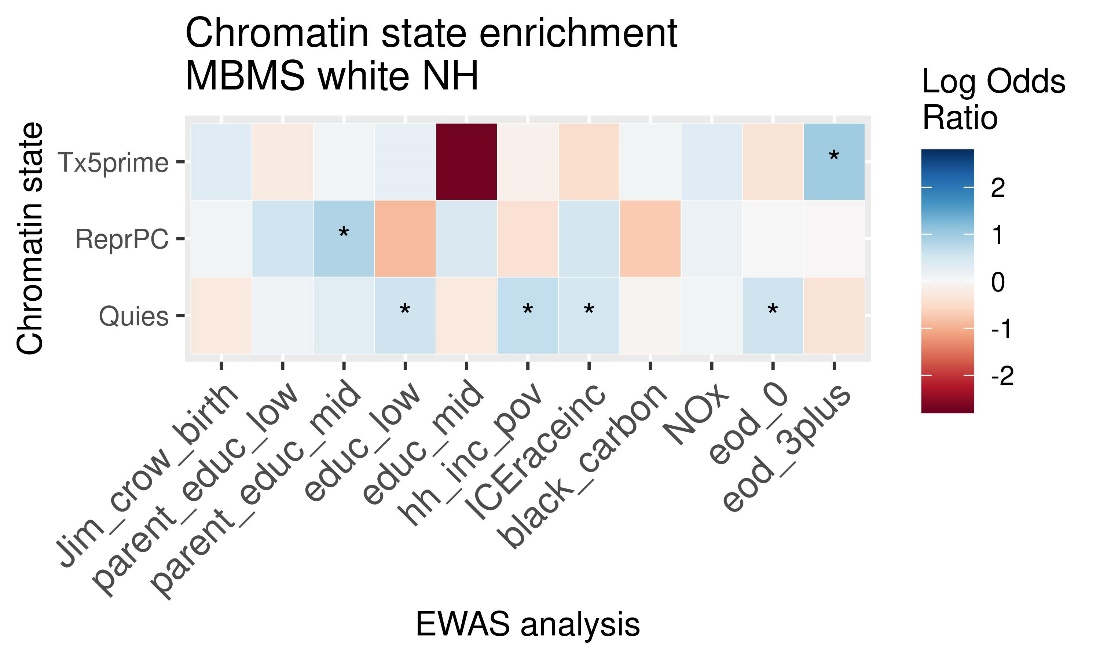


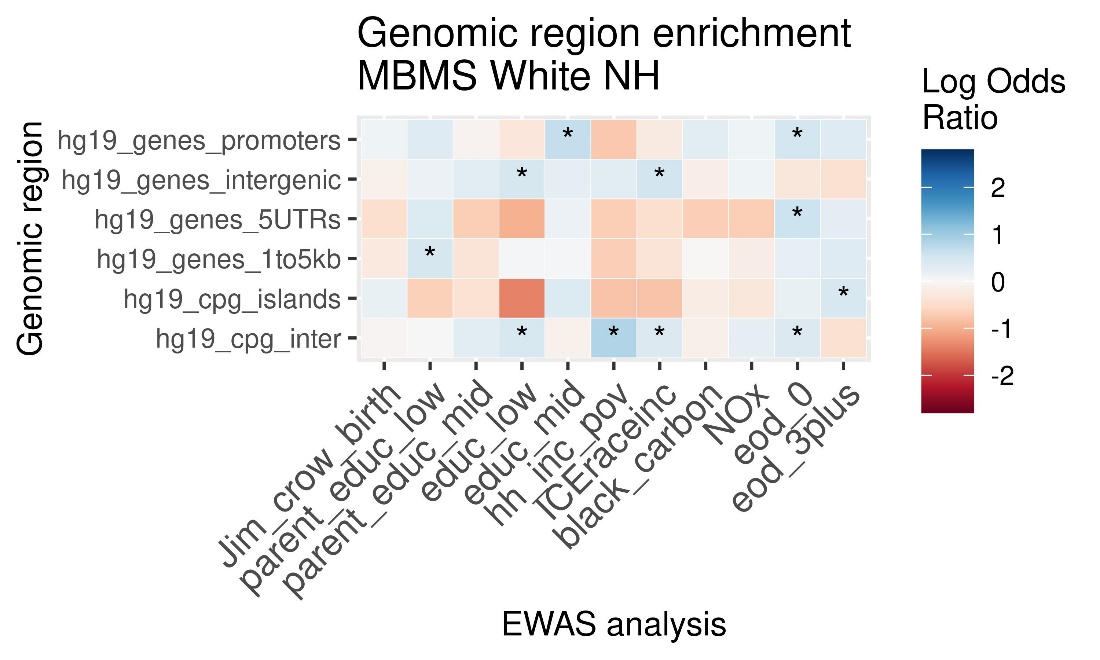


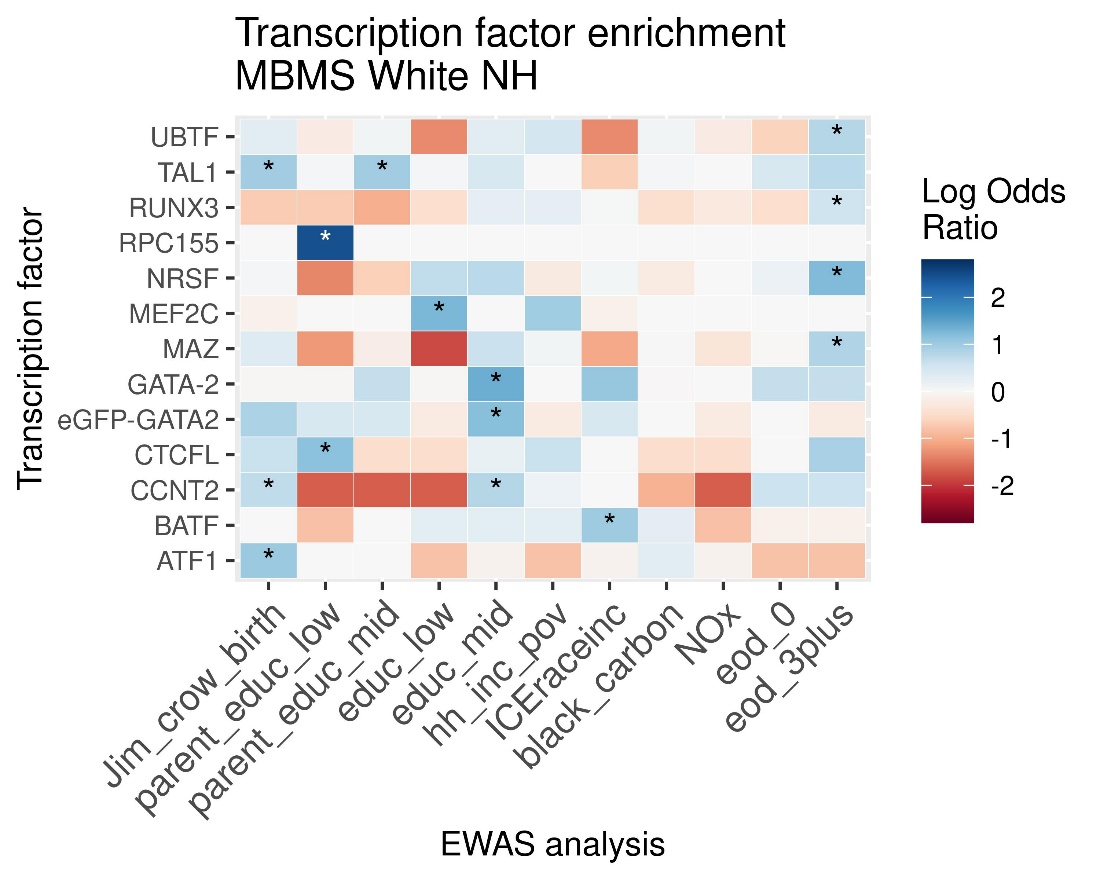


### MESA full cohort


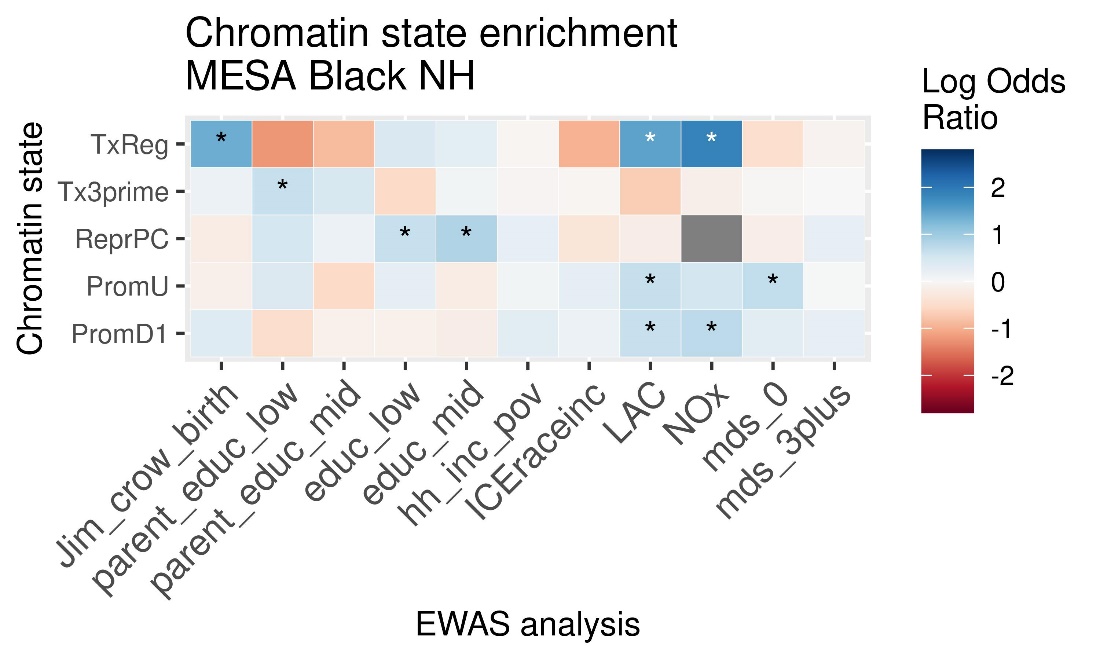


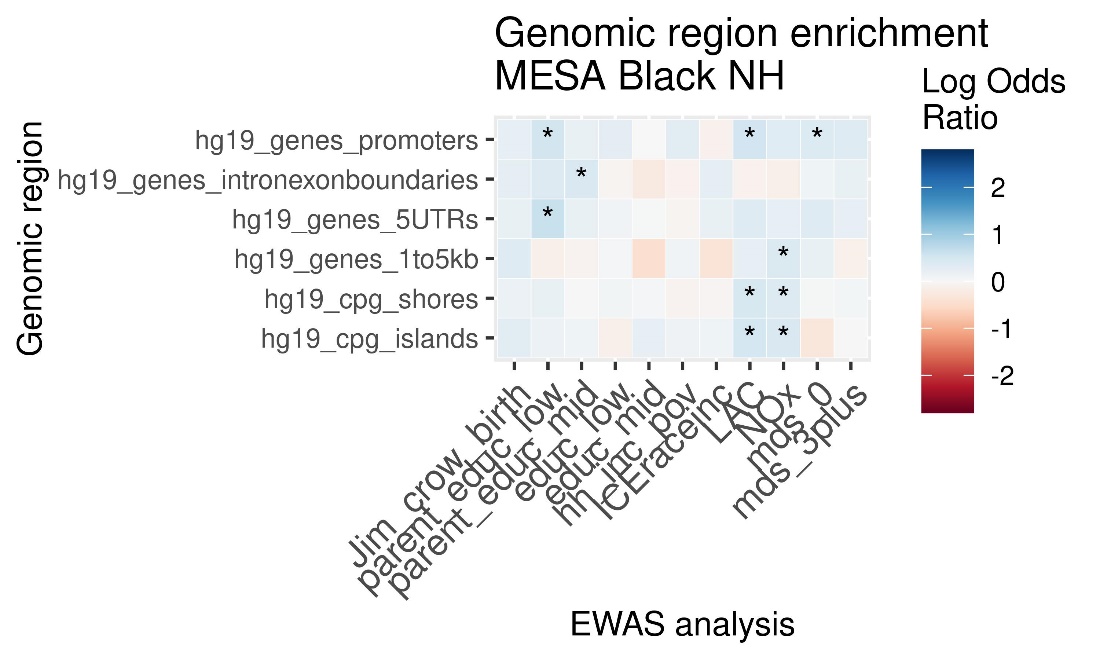


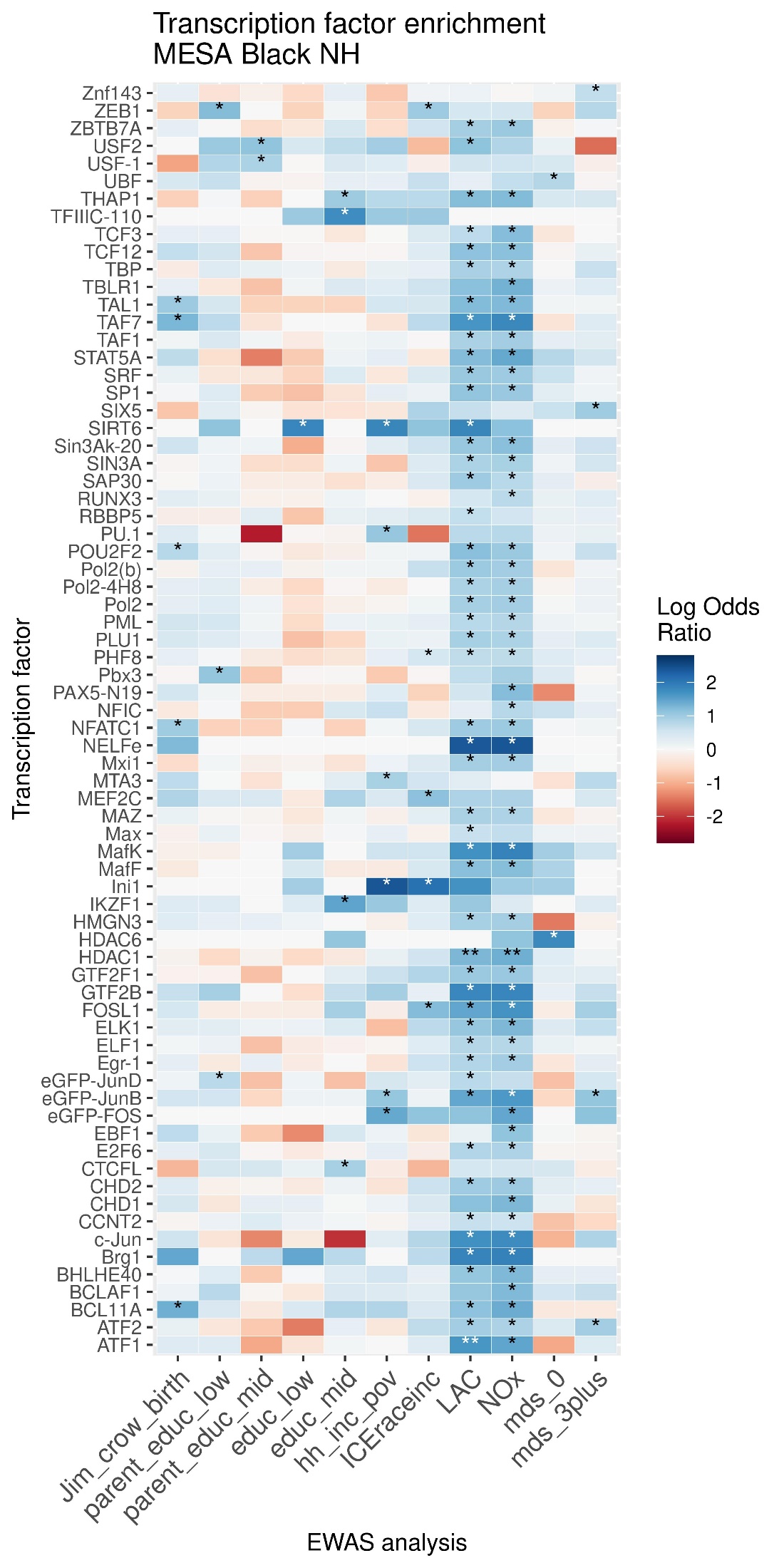


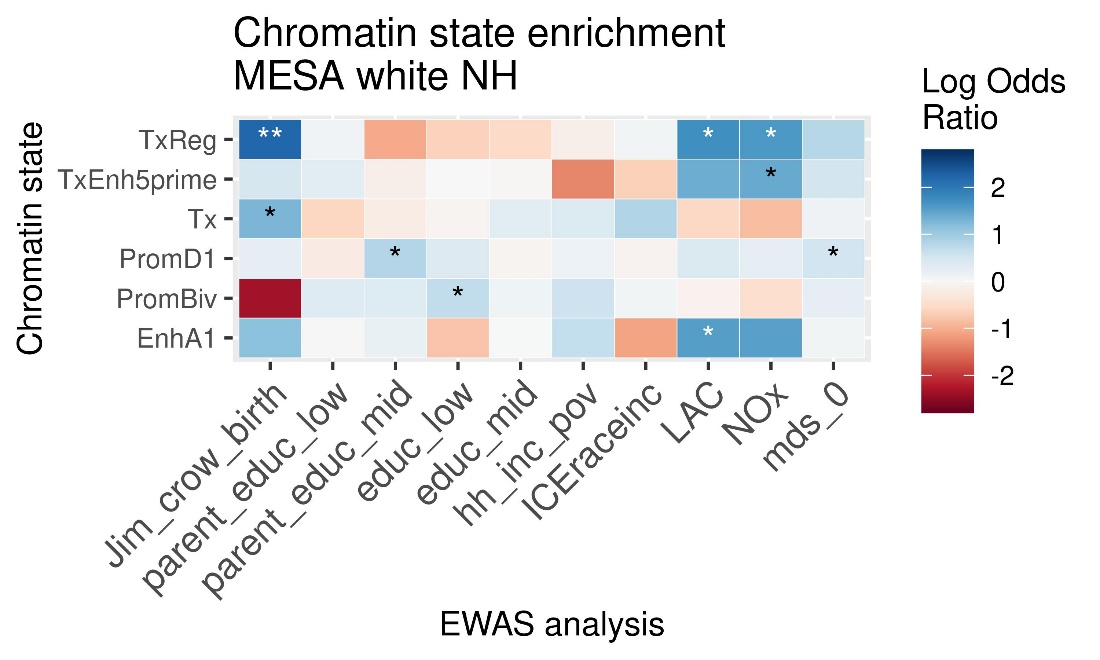

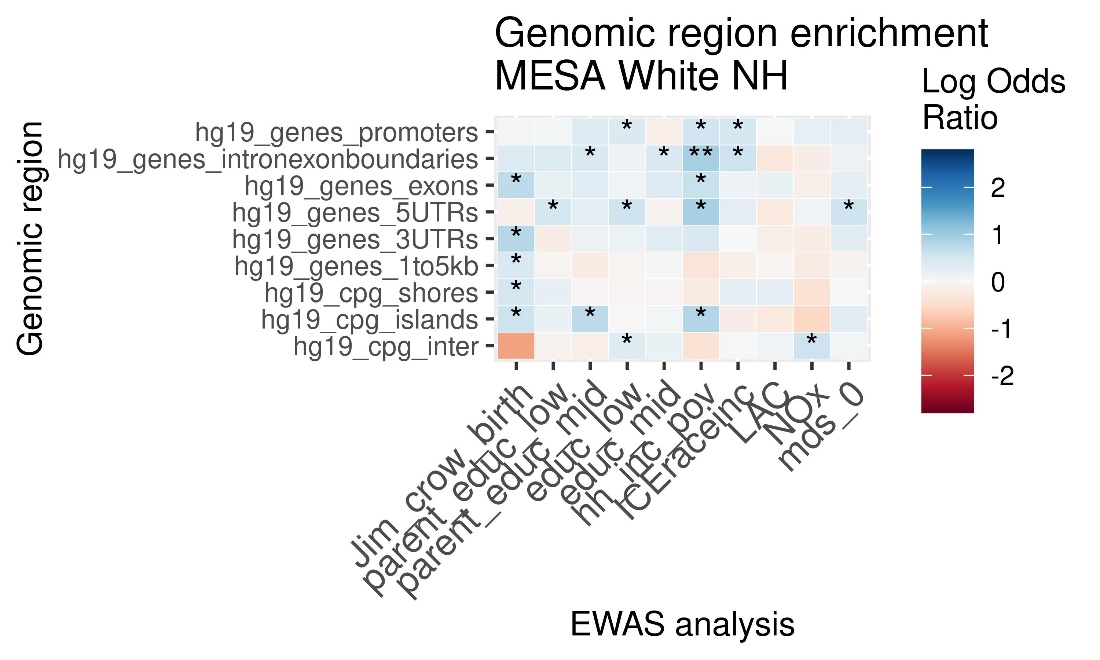

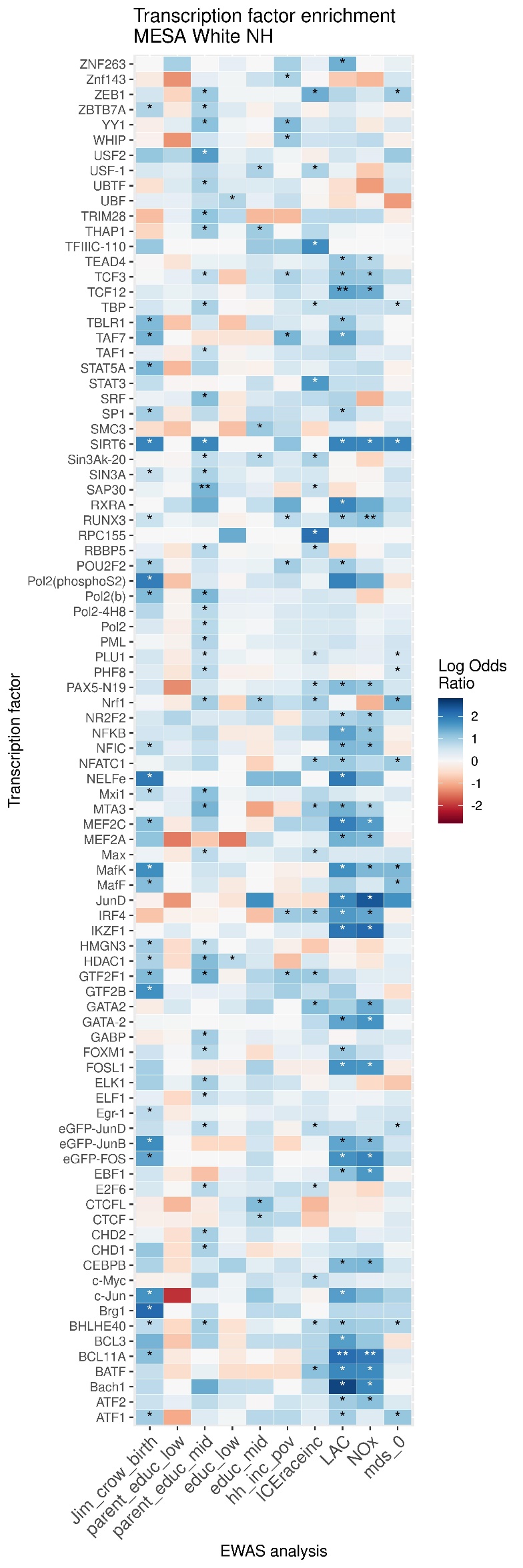


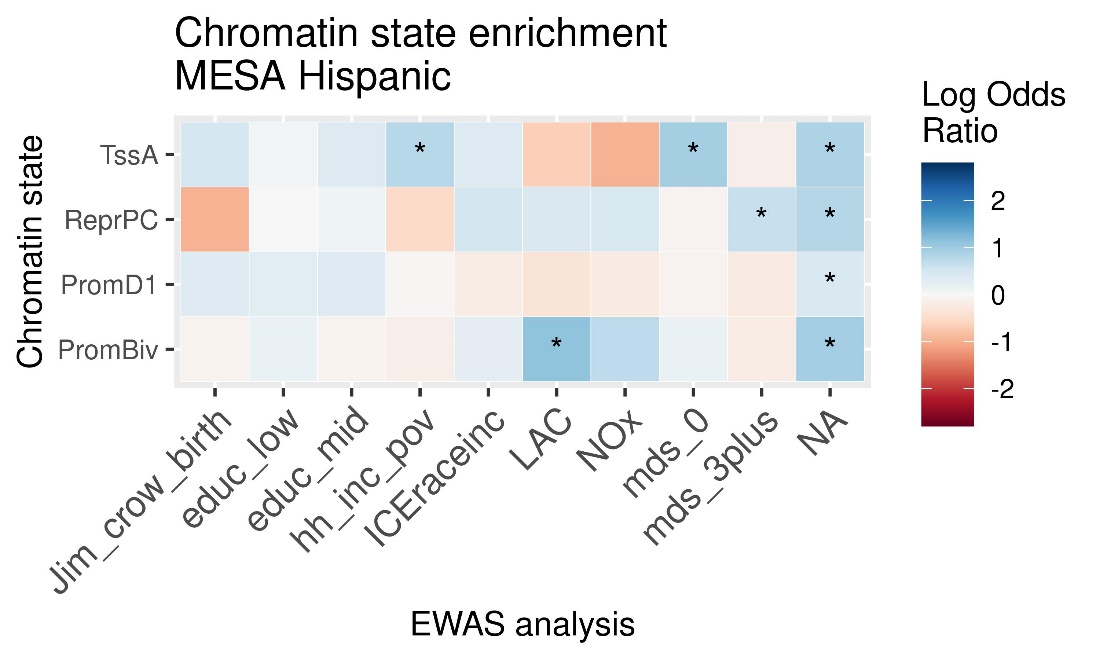

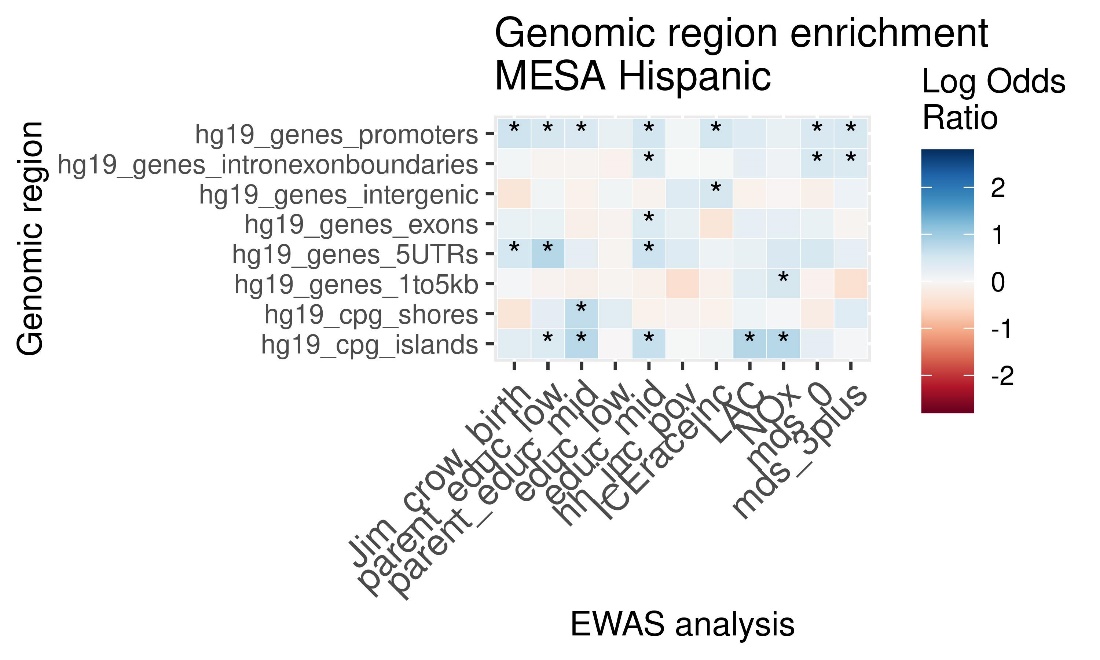

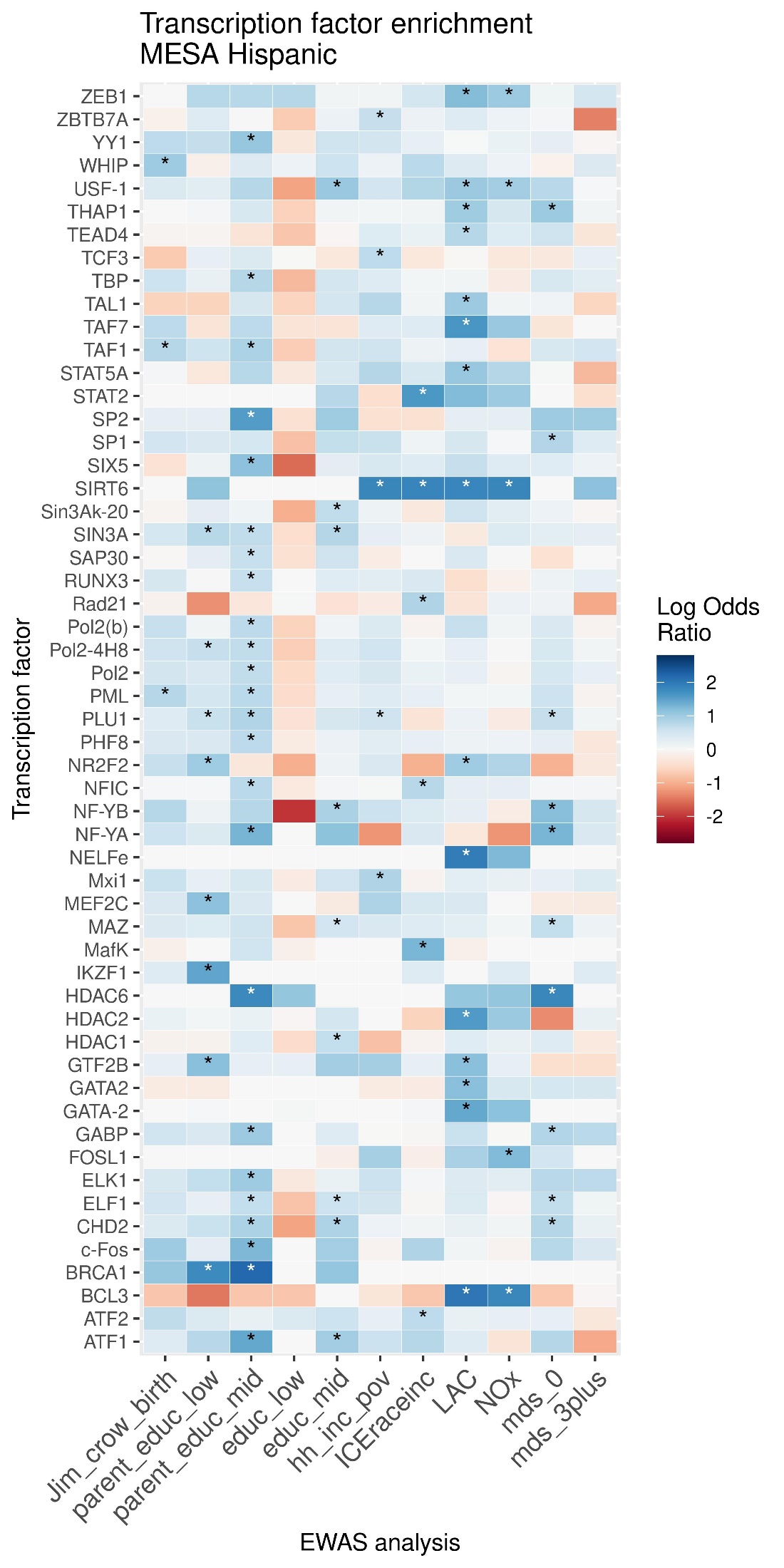


## MESA subgroup


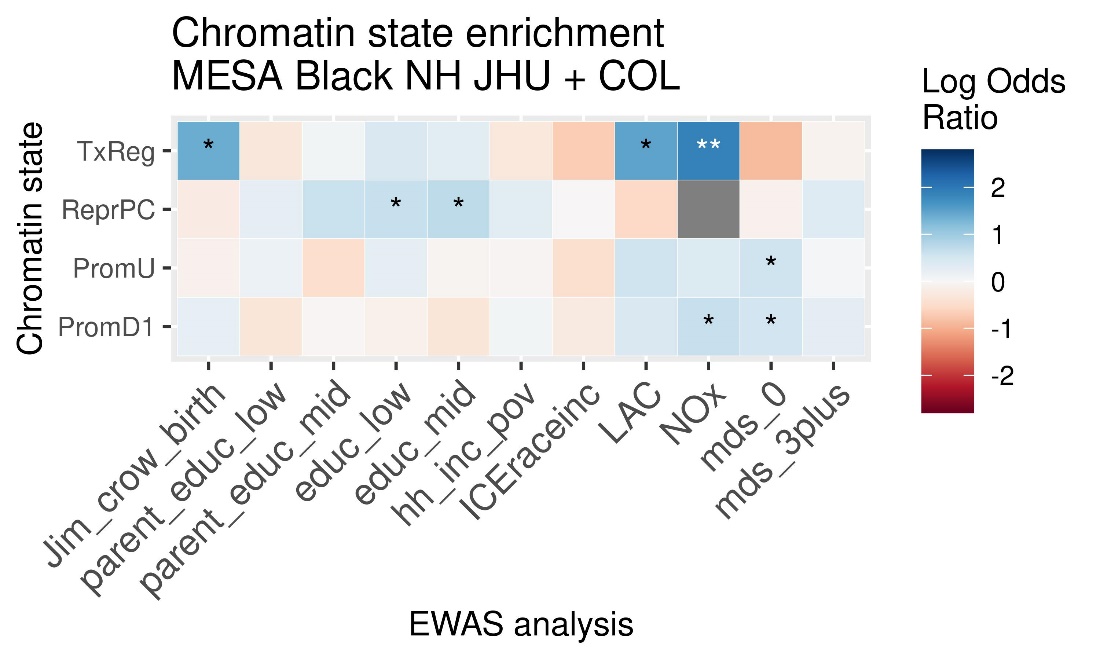


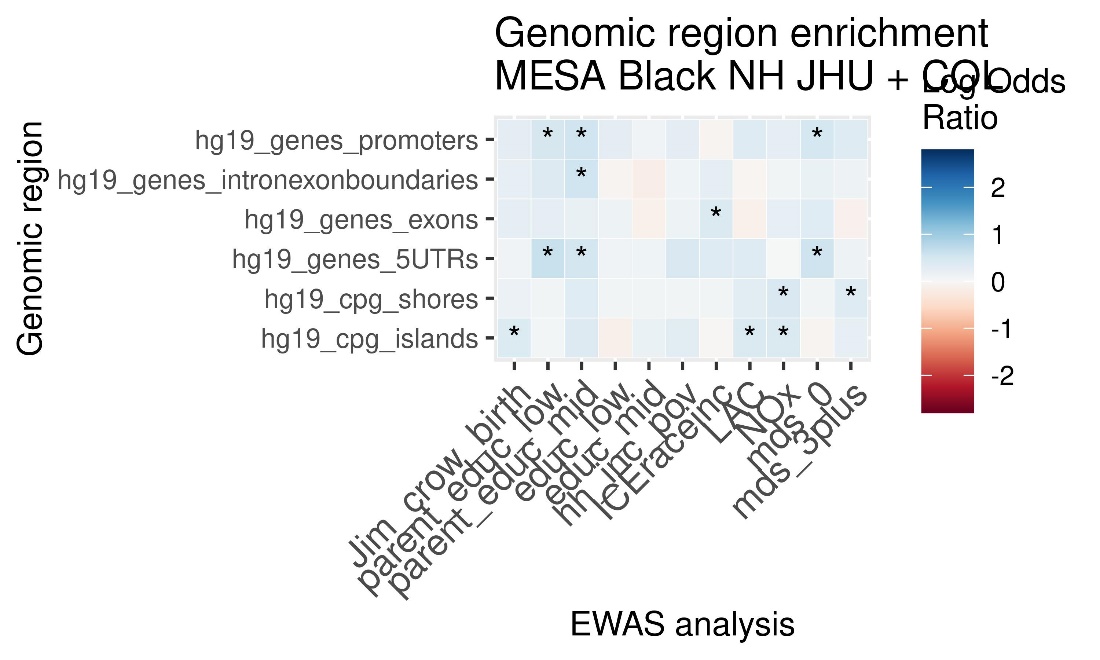


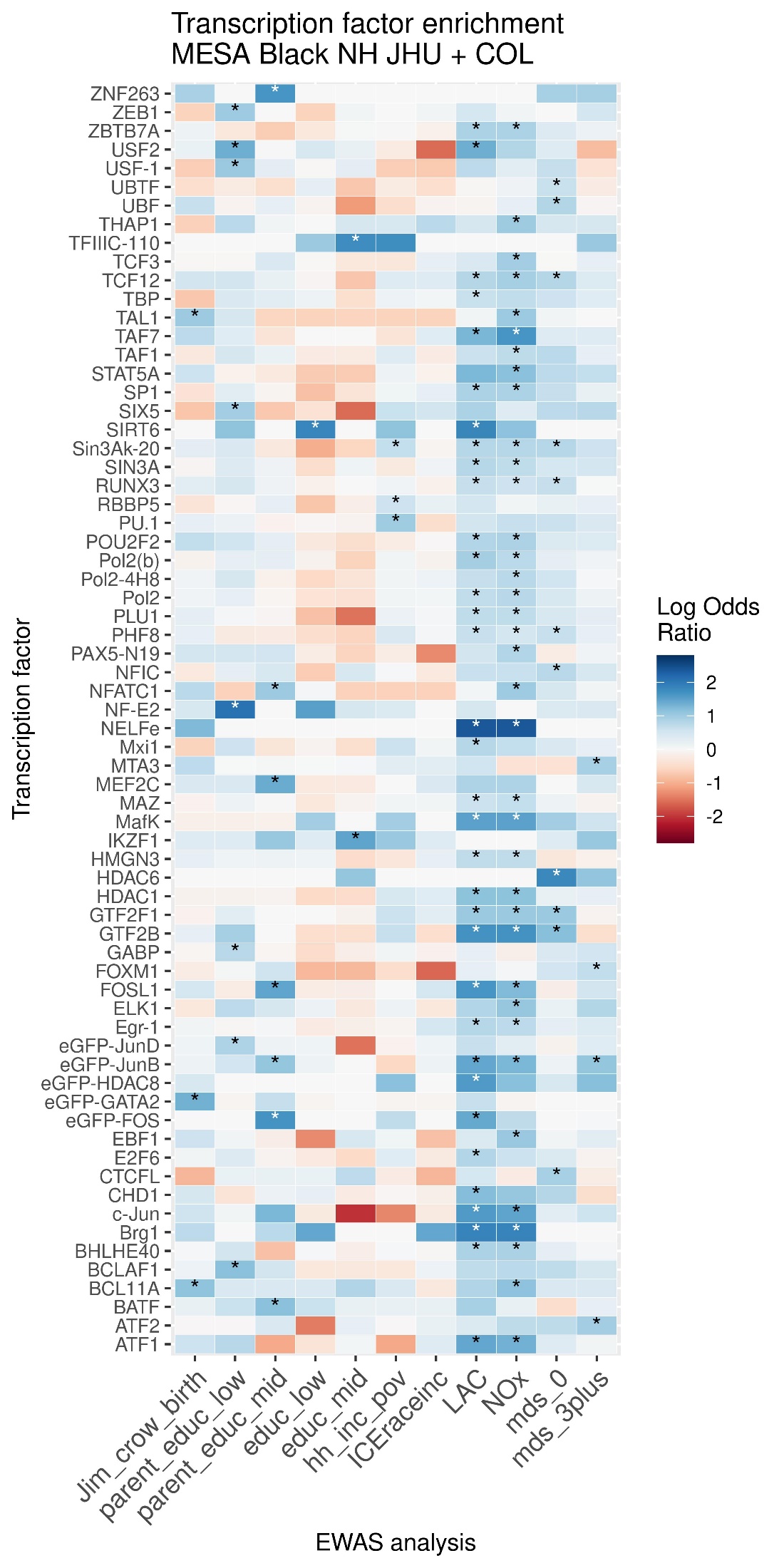


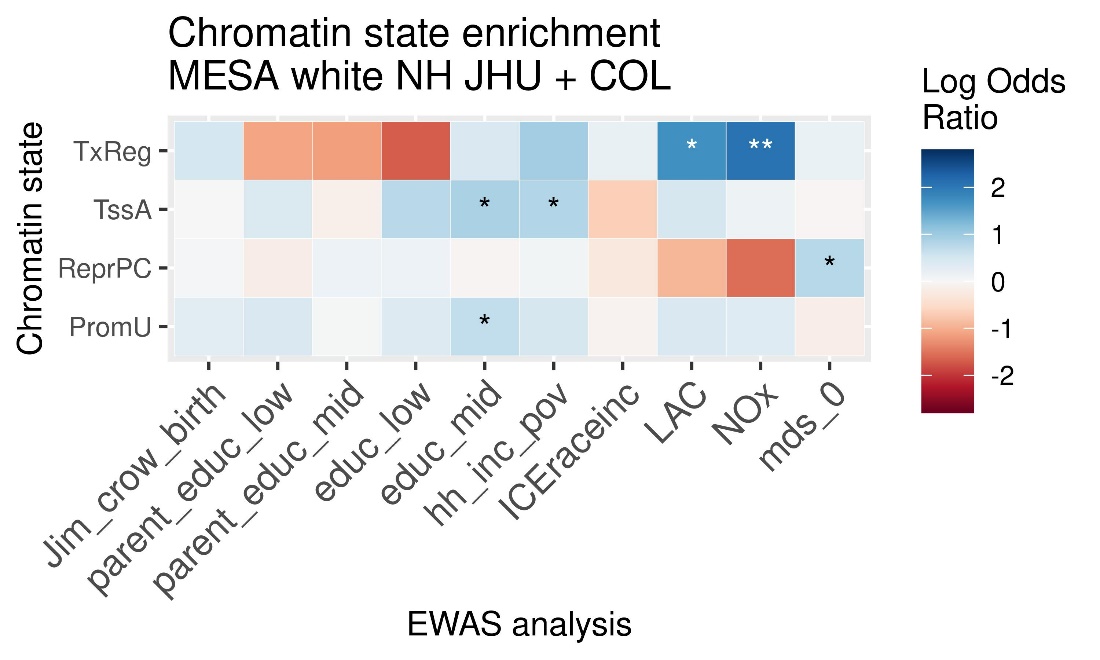


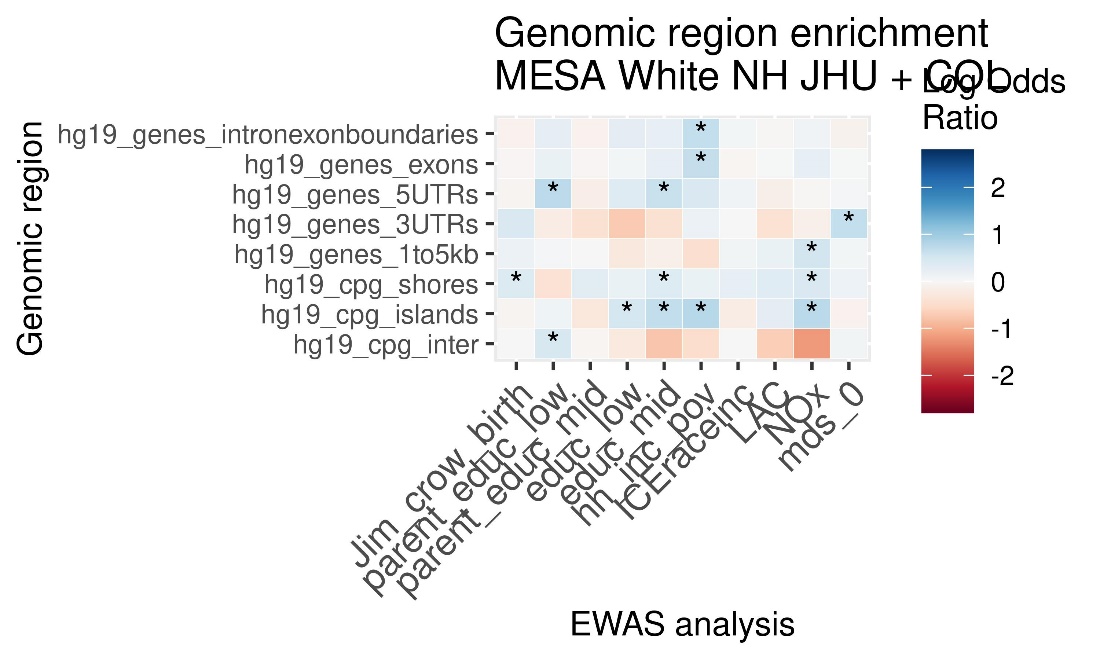


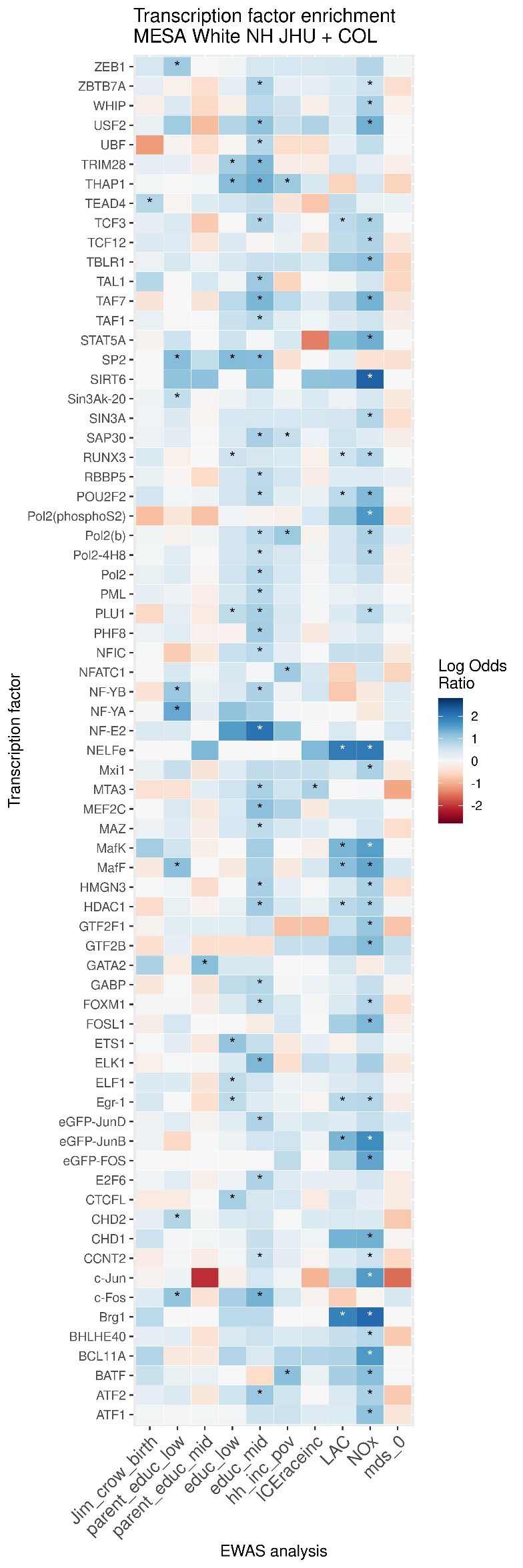


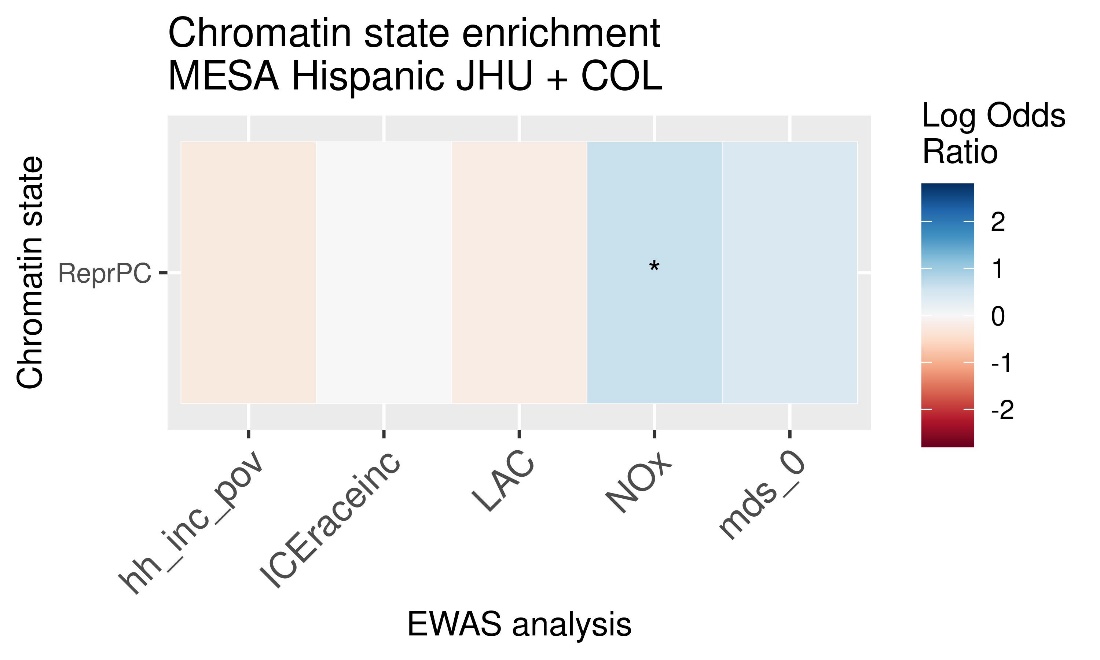


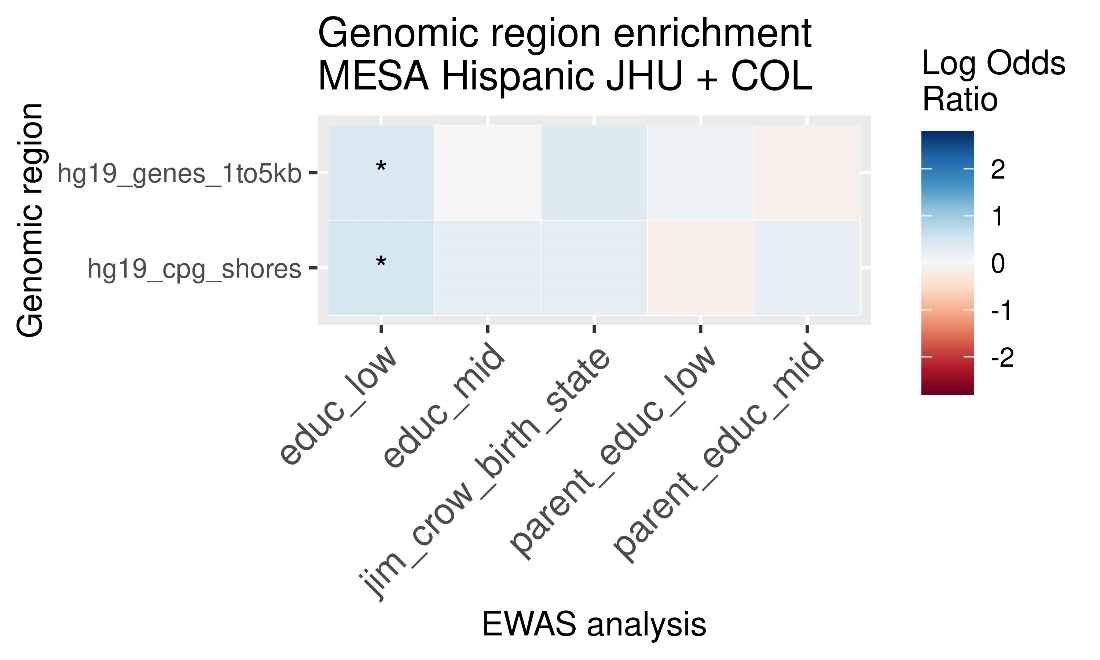


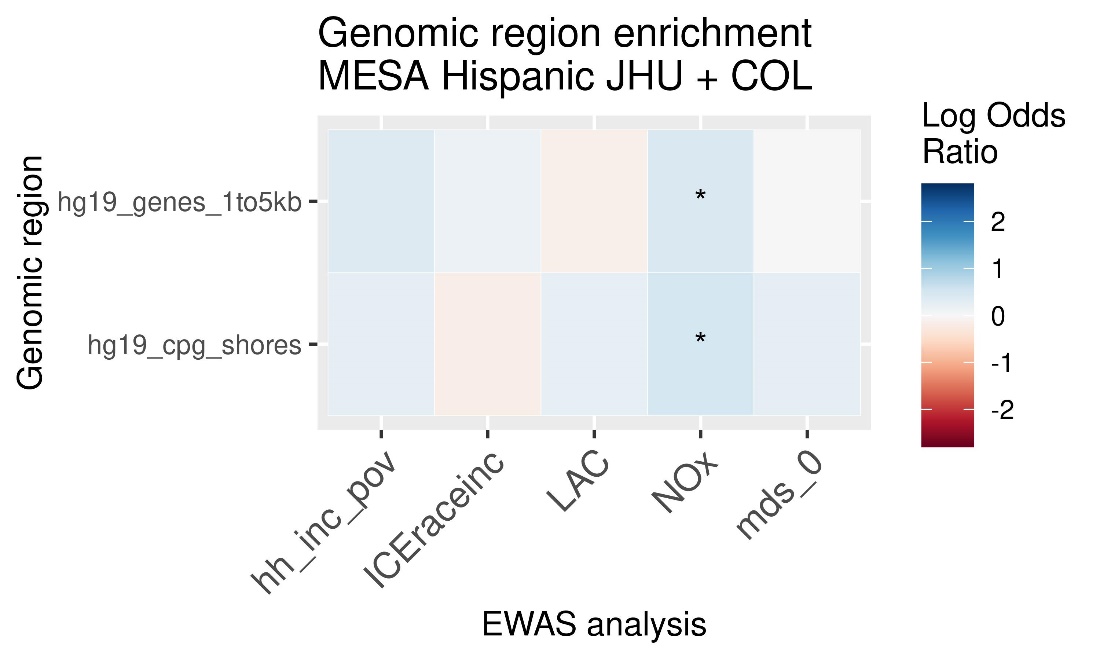


## MBMS meta-analysis


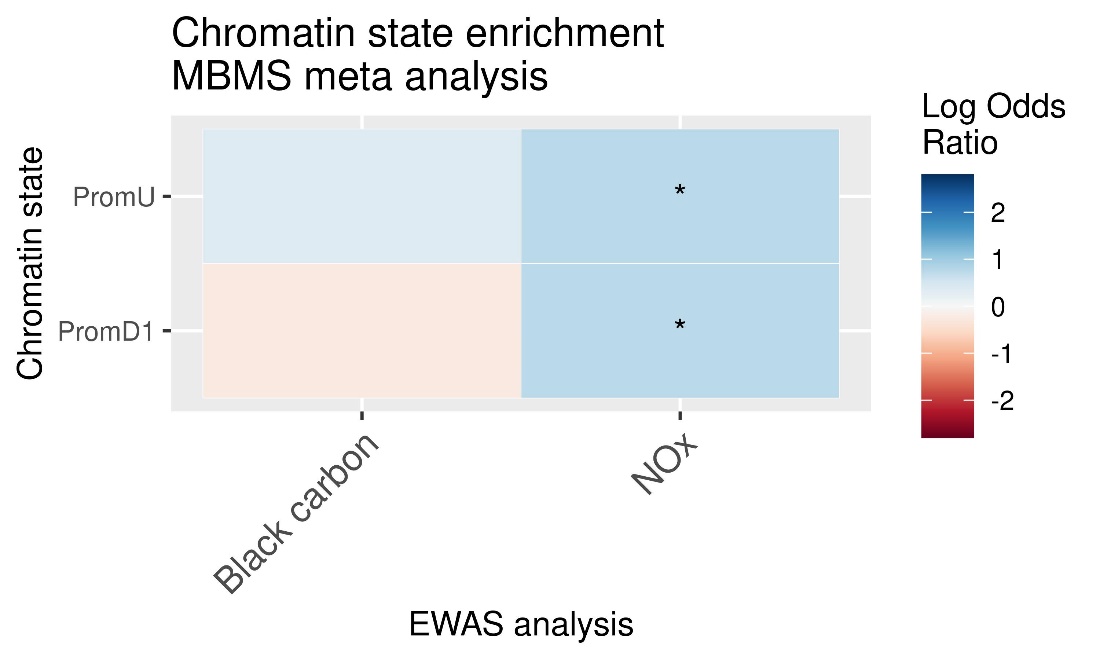


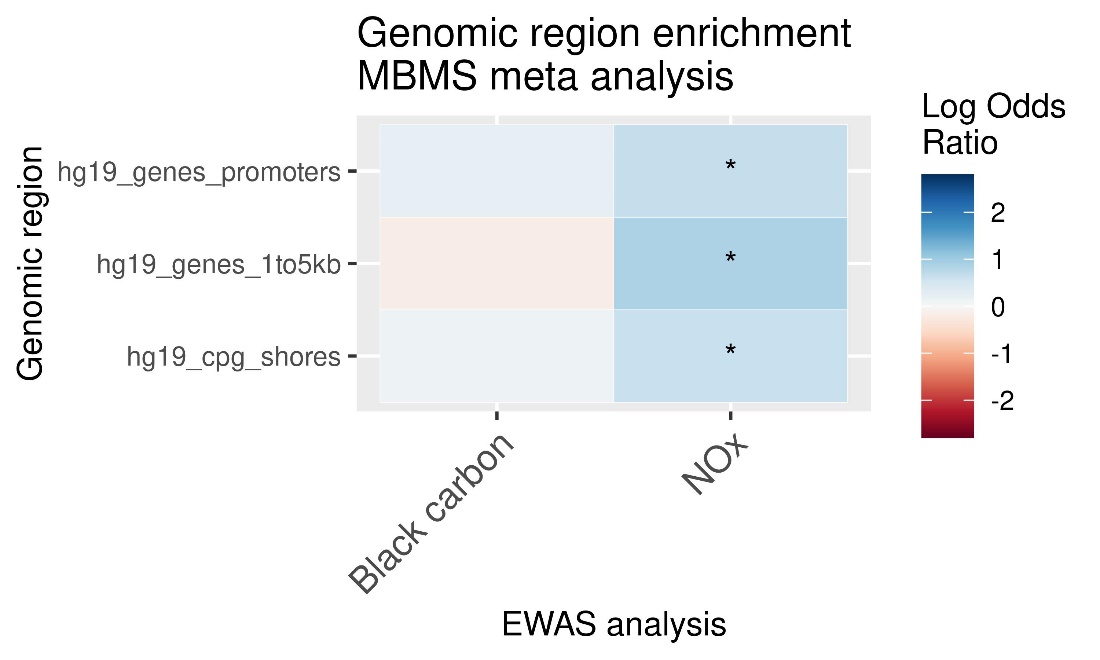


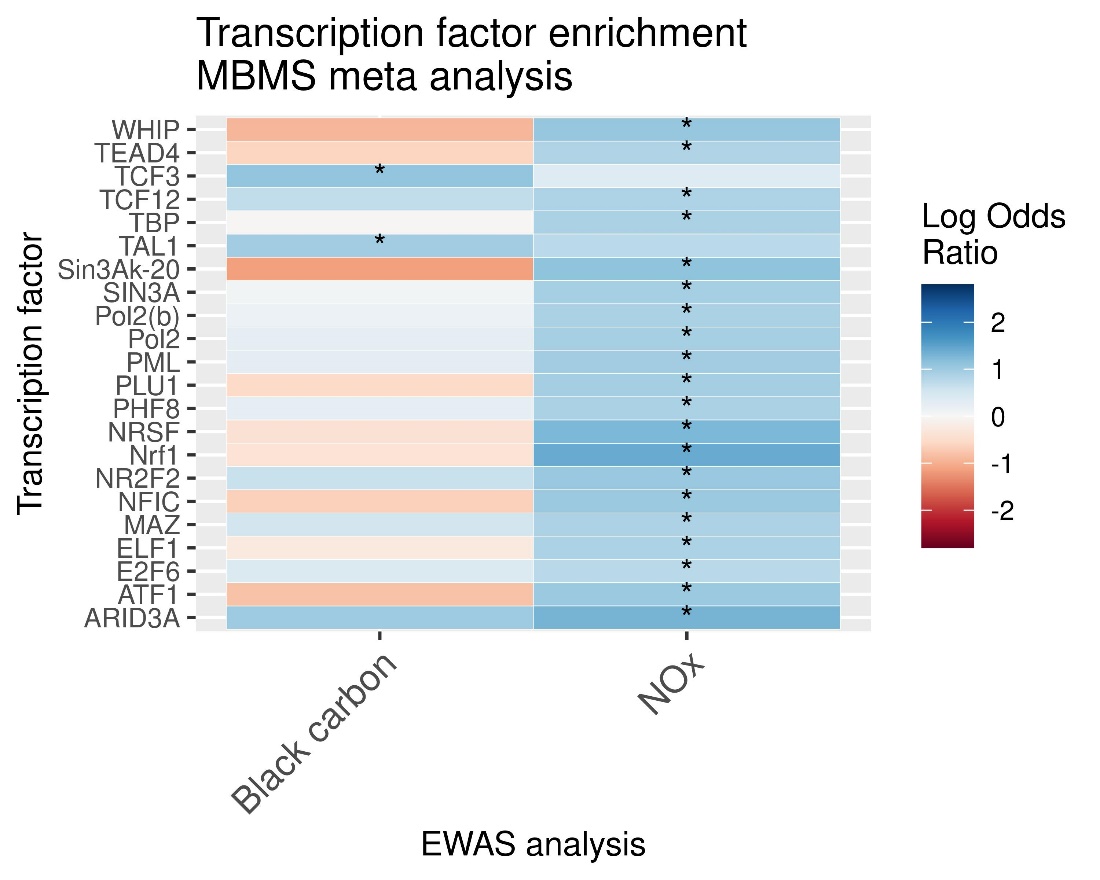


## Mesa meta-analysis (full cohort)


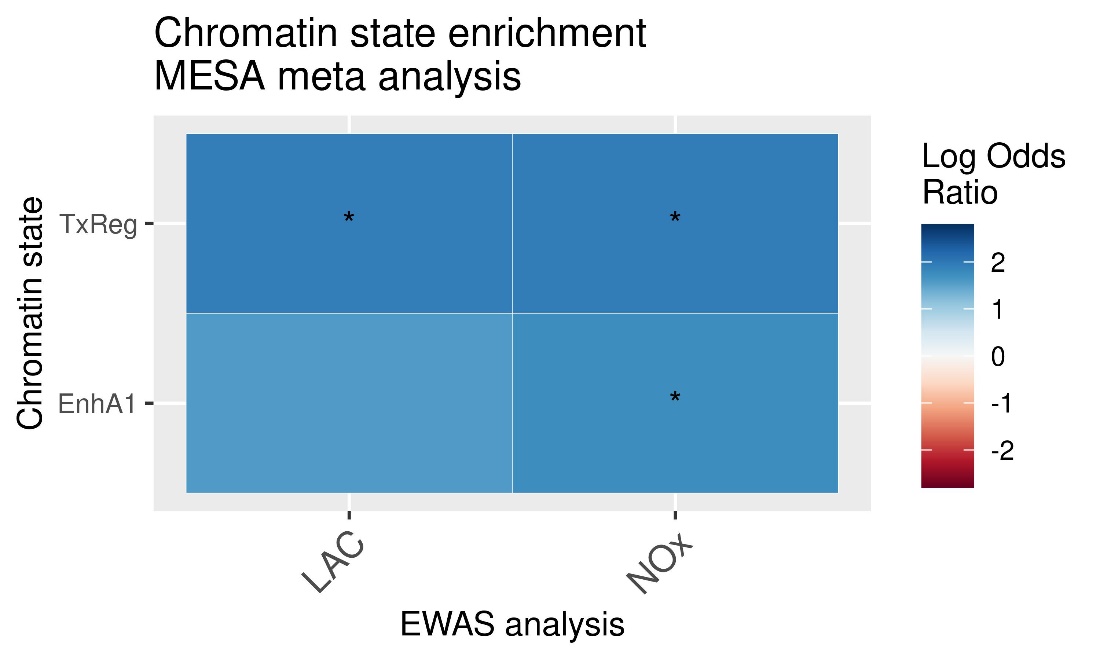


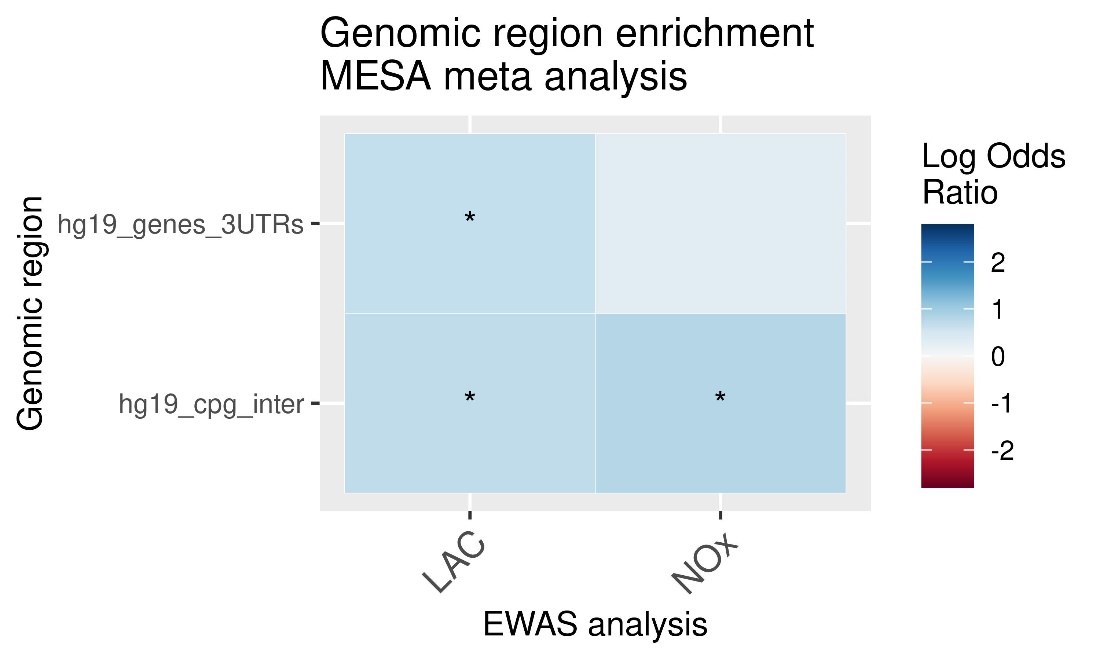


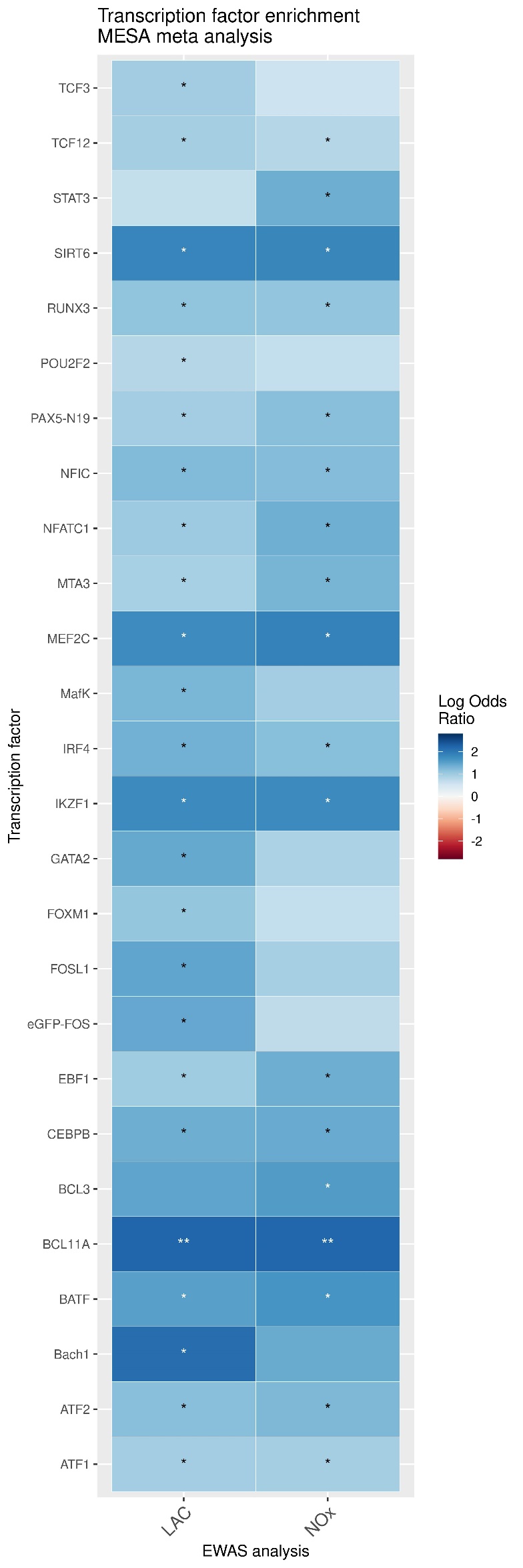


## MESA meta-analysis (subgroup)


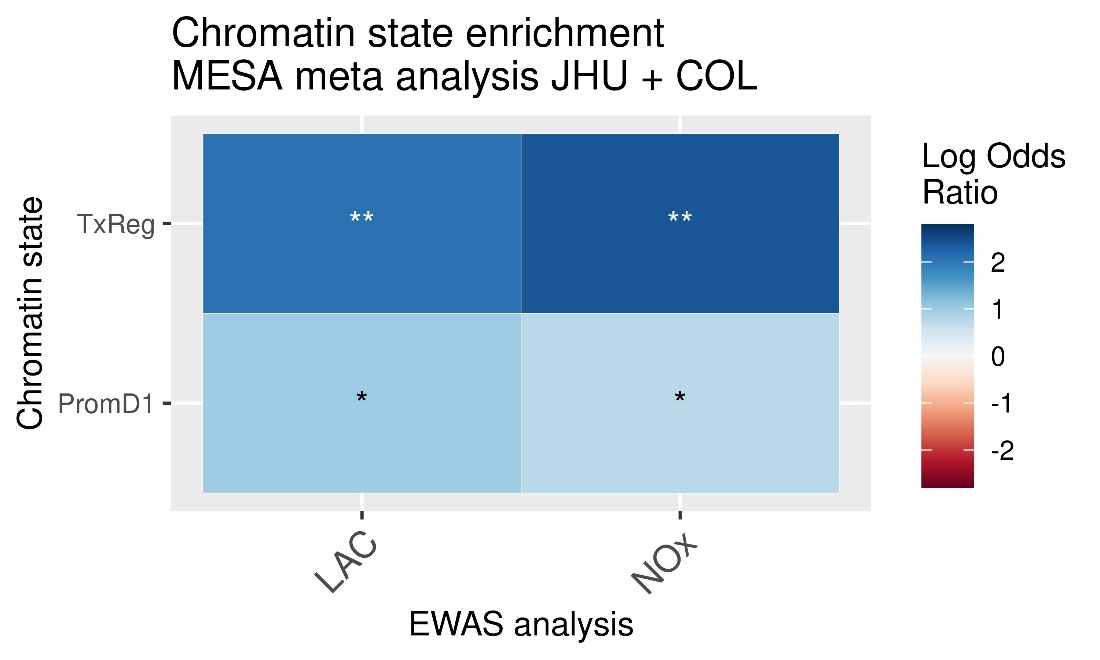


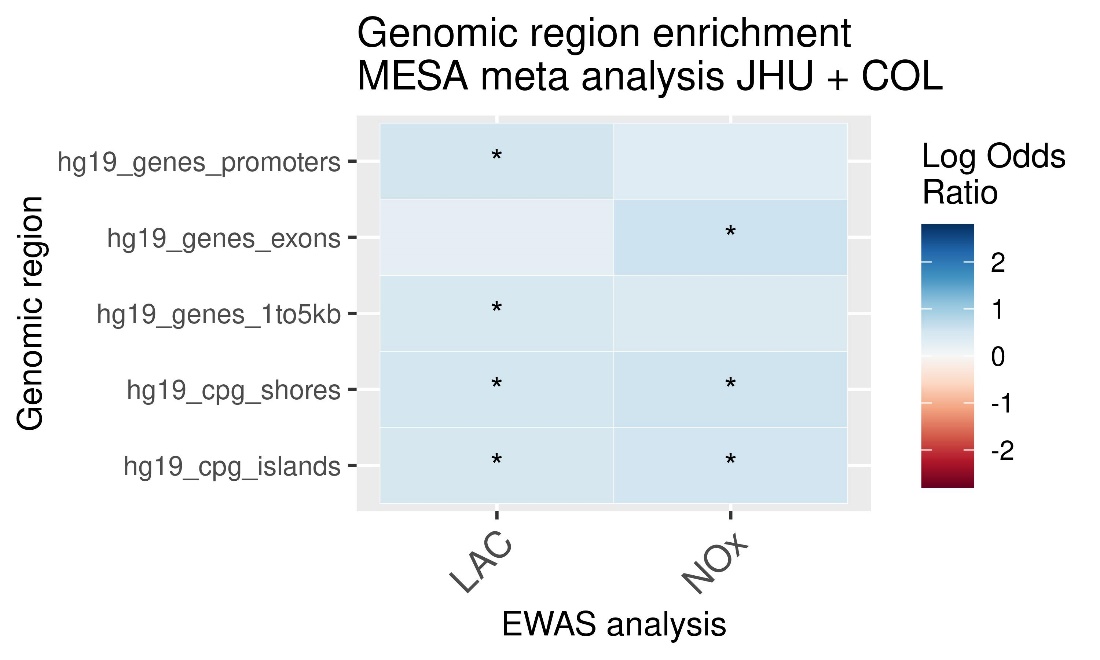


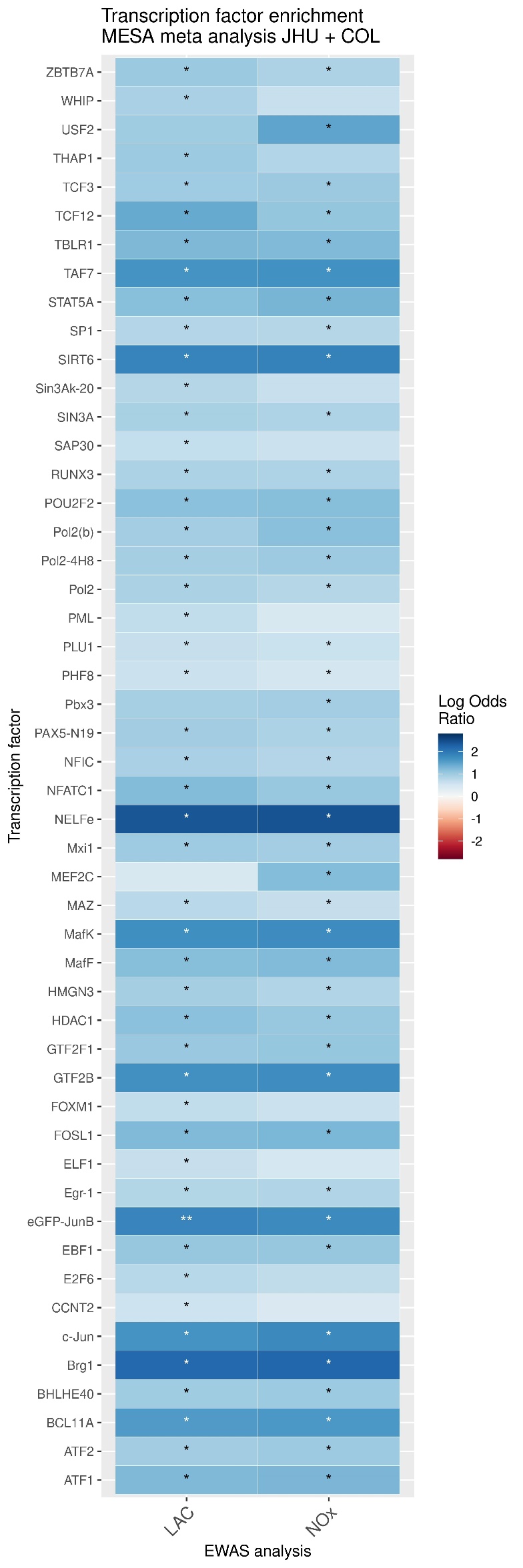

Supplement: Supplement 1 [file media-1.docx]
